# Improving ancient DNA read mapping against modern reference genomes

**DOI:** 10.1186/1471-2164-13-178

**Published:** 2012-05-10

**Authors:** Mikkel Schubert, Aurelien Ginolhac, Stinus Lindgreen, John F Thompson, Khaled AS AL-Rasheid, Eske Willerslev, Anders Krogh, Ludovic Orlando

**Affiliations:** 1 Centre for GeoGenetics; Natural History Museum of Denmark, University of Copenhagen, 5-7 Øster Voldgade, 1350, Kobenhavns K, Denmark; 2 The Bioinformatics Centre, Department of Biology, University of Copenhagen, Ole Maaloes Vej 5, 2200, Copenhagen, Denmark; 3 Applications, Methods and Collaborations; Helicos BioSciences, One Kendall Square Bldg 200LL, 02139, Cambridge, MA, USA; 4 Present address: NABsys Inc, 60 Clifford Street, 02903, Providence, RI, USA; 5 Zoology Department, College of Science King Saud University, P.O. Box 2455, Riyadh, 11451, Saudi Arabia

## Abstract

**Background:**

Next-Generation Sequencing has revolutionized our approach to ancient DNA (aDNA) research, by providing complete genomic sequences of ancient individuals and extinct species. However, the recovery of genetic material from long-dead organisms is still complicated by a number of issues, including *post-mortem* DNA damage and high levels of environmental contamination. Together with error profiles specific to the type of sequencing platforms used, these specificities could limit our ability to map sequencing reads against modern reference genomes and therefore limit our ability to identify endogenous ancient reads, reducing the efficiency of shotgun sequencing aDNA.

**Results:**

In this study, we compare different computational methods for improving the accuracy and sensitivity of aDNA sequence identification, based on shotgun sequencing reads recovered from Pleistocene horse extracts using Illumina GAIIx and Helicos Heliscope platforms. We show that the performance of the Burrows Wheeler Aligner (BWA), that has been developed for mapping of undamaged sequencing reads using platforms with low rates of indel-types of sequencing errors, can be employed at acceptable run-times by modifying default parameters in a platform-specific manner. We also examine if trimming likely damaged positions at read ends can increase the recovery of genuine aDNA fragments and if accurate identification of human contamination can be achieved using a strategy previously suggested based on best hit filtering. We show that combining our different mapping and filtering approaches can increase the number of high-quality endogenous hits recovered by up to 33%.

**Conclusions:**

We have shown that Illumina and Helicos sequences recovered from aDNA extracts could not be aligned to modern reference genomes with the same efficiency unless mapping parameters are optimized for the specific types of errors generated by these platforms and by *post-mortem* DNA damage. Our findings have important implications for future aDNA research, as we define mapping guidelines that improve our ability to identify genuine aDNA sequences, which in turn could improve the genotyping accuracy of ancient specimens. Our framework provides a significant improvement to the standard procedures used for characterizing ancient genomes, which is challenged by contamination and often low amounts of DNA material.

## Background

Over the last three decades, ancient DNA (aDNA) research has demonstrated that genetic material can survive for up to several hundreds of thousand years in fossil materials [1], such as bones, teeth, soft tissues, hair, coprolites and soil sediments [2]. One successful application of aDNA has been to chart the diversity of paleo-communities through time, revealing how major environmental crises have transformed the composition of past ecosystems [3] and have impacted the past demographic dynamics of several megafauna species until their extinction [4,5]. Ancient DNA has also been used to reveal the evolutionary origin of human populations [6-8] and to investigate our relationships with archaic hominins [9-11]. The sequencing of nucleic acids from ancient pathogens, including the 1917 influenza virus responsible for 20–40 millions of human deaths worldwide [12], has provided insights into the molecular mechanisms involved in virulence with potential applications in medicine and epidemiological surveys.

The recent advent of Next-Generation Sequencing (NGS) technologies heralded a revolution in aDNA research, by providing access to the complete genomic sequences of ancient individuals and extinct species [7-9,11,13]. Most of the genetic information available at the genome-wide level has been generated using second-generation platforms, first on 454 Roche genome sequencers [13,14], then on Illumina platforms [7-9,11]. Common to all second-generation platforms is the need for building and amplifying DNA libraries before sequencing, leading to a skewed representation of the underlying sample. Recently, the first successful application of library-free third-generation sequencing of aDNA templates has been reported from a Pleistocene horse bone using the Helicos true Single Molecule DNA Sequencing platform (tSMS) [15,16]. Of note, the different sequencing platforms available exhibit specific error profiles, with tSMS showing higher error rates than Illumina sequencing overall and a prevalence of insertions/deletions over nucleotide misincorporations [17]. In addition, specific sources of bias have been observed with tSMS, mostly as a result of the poly-A tailing and blocking reactions that are performed before sequencing and that skew the base composition of sequencing reads [15]. Adding to the DNA damage induced nucleotide misincorporations that are typical of ancient templates, these specificities could limit our ability to map sequencing reads against modern reference genomes and therefore limit our ability to identify genuine endogenous tSMS reads, reducing the efficiency of shotgun sequencing.

In this study, we explore different strategies for improving the mapping of sequencing reads against modern reference genomes, in order to improve the identification of endogenous aDNA fragments. We take advantage of two sets of sequence data generated from two Pleistocene horse bone DNA extracts on Illumina GAIIx and Helicos genome sequencer platforms [15,16]. Semi-global aligners, such as the Burrows Wheeler Aligner (BWA) [18] have been developed for mapping undamaged sequencing reads**,** generated from modern extracts**,** using platforms with low rates of indels and sequencing errors. To see if performance could be improved for aDNA, we have modified the default alignment parameters to improve mapping sensitivity at acceptable run-times. Taking advantage of the fact that nucleotide misincorporations resulting from *post-mortem* damage preferentially cluster at sequencing termini [15,19], we further test if systematic trimming of likely damaged positions at read ends could improve overall read mapping quality and thereby lead to the recovery of additional genuine aDNA fragments. We examine different methods designed to identify human contamination and their respective consequences in estimating endogenous sequence contents. We show that combining the different approaches can increase the number of high-quality endogenous hits recovered by up to 33.4% when mapping tSMS reads against modern reference genomes. The fraction of endogenous Illumina reads identified is less sensitive to the mapping strategy. Overall, we present a series of approaches that could substantially improve the ability to identify the true fraction of endogenous reads when shotgun sequencing aDNA with Illumina and Helicos next-generation sequencing platforms.

## Results and discussion

### Overall strategy

To optimize the performance of alignment using BWA, the use of a seed region (option -l), the prohibition of indels close to read ends (option -i), the maximum number of gap opens (option -o) and gap penalties (options -O and –E), and the overall number of differences tole-rated in the alignment (option -n) were examined. Illumina and Helicos sequencing reads were mapped against both the horse (*Equus caballus*) and the human reference genomes and high-quality endogenous hits were defined as hits found to map to a unique location in the horse genome with mapping qualities equal or higher than 25 while showing no high-quality hit to the human reference genome. The latter was introduced as a conservative approach to limit the fraction of spurious hits that could result from chance alignments of reads of exogenous origin to the horse genome. Additionally, the level of spurious hits recovered with the different sets of mapping parameters was monitored by the fraction of high-quality hits recovered when performing alignments against the non-mammalian chicken genome (galGal3).

As endogenous aDNA reads have been shown to exhibit specific miscoding lesions that could be used as molecular signatures of *post-mortem* DNA damage, nucleotide misincorporation patterns [20] were used to assess the quality of the extra hits recovered with the different mapping strategies explored. These patterns mainly result from *post-mortem* deamination of cytosine residues into uracil residues, and for Illumina reads consist of an increase of C → T misincorporations at the 5’-ends of sequencing reads paralleled by an increase in G → A misincorporations at 3’-ends [10,19,21]; for Helicos tSMS reads, the deamination of cytosine residues results in an increase in G → A mismatches at 5’-ends of sequencing reads [15]. Finally, we validated the optimal set of mapping parameters for the Helicos tSMS platform using a simulation approach, showing that the suggested parameters preferentially lead to alignments at the correct location, while not significantly inflating the rate of spurious alignments.

### Seed region

By default, BWA uses the first 32 nucleotides of query sequences as a seed region, allowing at most two mismatches in this region in order to reduce the overall runtime of the alignment (the seed is disabled for reads that are shorter than the length of the seed region). Unless specific enzymatic treatments are used (e.g. as part of the library building protocol; see [22]), aDNA reads exhibit high rates of mismatches at 5’-termini due to the prevalence of *post-mortem* cytosine deamination at overhanging ends. The prevalence of such damage-related mismatches in the seed region could limit the ability of seed-based mapping strategies to identify genuine but highly damaged hits for both Illumina and Helicos platforms. In addition, Helicos tSMS reads starting with a minimum of two thymine residues are trimmed post-sequencing, as these could have resulted from the sequencing of the poly-A tail added early in the template preparation procedure. This has been shown to increase mismatch rates from thymine residues to any other nucleotide base at the 5' read termini [15], which again would increase the number of mismatches present at 5’ termini and could further limit the performance of seed-based mapping strategies. Therefore, we examined whether significant yields of endogenous hits could be recovered by disabling the seed region.

**Figure 1:**
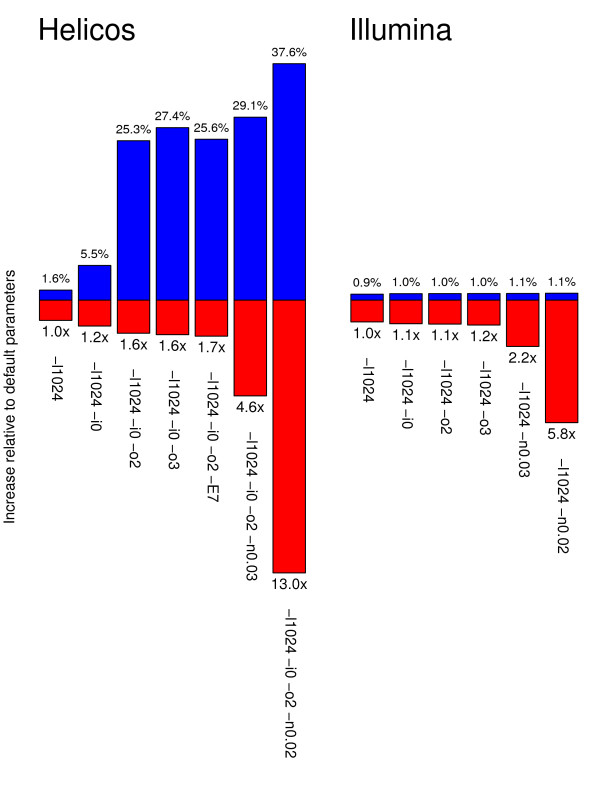
**Exploring the effects of different sets of mapping parameters on BWA performance and runtime.** Sequencing reads recovered from the sample showing infinite radiocarbon date were aligned using different combinations of mapping parameters using the BWA aligner. Reads were considered of high-quality when mapping uniquely to the EquCab2 genome but not against the human genome (assembly hg19) and showing mapping qualities of at least 25. For Illumina, positive hits were filtered for PCR duplicates (see Methods). Performance and runtime are estimated with reference to the standard default parameters. *Left*: Helicos tSMS reads. *Right*: Illumina reads.

Disabling the seed region yielded a 0.9% increase in the total number of high-quality hits for Illumina sequences, while doubling the runtime (Figure 1). A higher relative gain of 1.6% was observed for Helicos sequences, at similar runtime costs (Figure 1). Interestingly, the new population of hits identified for both platforms shows an excess of cytosine deamination related mismatches at 5’-ends (C → T for Illumina and G → A for Helicos, Additional file 1: Figure S1, S2), suggesting that these most likely consist of DNA templates with 5'-overhangs, that were lost due to the use of a seed region. Importantly, nucleotide misincorporations related to cytosine deamination were found to dominate mismatch patterns over the full length of sequencing reads, confirming that the new hits identified consisted of reads of ancient origin, as cytosine deamination has been shown to be the most prevalent form of *post-mortem* damage [19,22-25]. This is further attested by the read size distributions of the new hits identified, which are enriched in reads of at least 38 nucleotides (Helicos) or 64 nucleotides (Illumina), which are very unlikely to be the result of chance alignments. This is confirmed by a very low number of matches when we map against the chicken reference genome (213–285 extra hits in contrast to 17,430-57,747 extra hits when mapping against the horse reference genome; see Additional file 1: Figure S3–S5 for related size distributions). 

### Indels at read ends

By default, BWA prohibits indels within the first and last five positions in alignments. This behavior appears suitable for platforms that produce low levels of spurious insertions and deletions during sequencing, such as the Illumina platforms. However, for tSMS and more generally third-generation sequencing, the most common type of sequencing errors are indels, particularly deletions [26]. Reads with indels near termini would consequently have an increased number of mismatches when aligned to a reference sequence, which will most likely result in a significant loss of positive hits. As expected, disabling the seed and allowing for indels near read termini improved the amount of high-quality hits recovered on the Helicos tSMS platform (+5.5%) while only slightly increasing the runtime cost (Figure 1). A similar trend was found using our simulation framework, where a higher fraction of simulated horse tSMS reads could be mapped at the right location of the genome, albeit with lower gains in extra-hits ( Additional file 1: Figure S6). The extra hits identified from tSMS sequences were found to exhibit the nucleotide misincorporation pattern characteristic of ancient templates, with an excess of gaps opened at alignment termini ( Additional file 1: Figure S7). In contrast to what is observed for Helicos data, no major improvement in the fraction of hits identified was detected for Illumina reads (Figure 1). On the contrary, disabling the seed and allowing for indels at read termini resulted in spurious nucleotide indel patterns, especially at 3’-ends, most likely resulting from local misalignments due to the addition of indels instead of expected substitutions.

### Adjusting gap openings and penalties

By default, BWA uses a high penalty for opening gaps (11) and a significantly lower penalty for extending gaps (4), though still higher than the penalty used for a mismatch (3). In addition, only one indel can be opened per alignment during the alignment phase. This appears to be an optimization towards sequencing platforms that produce few indels, such as the Illumina platform. However, indels, and particularly deletions, represent the most frequent type of sequencing error on Helicos platforms, with overall rates per base estimated at around 5% [17]. For the size range relevant in this case study (25–57 nucleotides), Helicos tSMS reads would therefore be expected to show a minimum of 1–2 indels on average, suggesting that standard parameters might lead to suboptimal performance. We therefore tested if extra high quality hits could be recovered for Helicos tSMS sequences, by increasing the maximum number of indels allowed to two. This was tested together with disabling seed and allowing indels at read termini, in agreement with the findings presented above.

Allowing more gap openings was found to have a dramatic influence on mapping results at relatively low runtime costs, with extra-gains of high-quality reads spanning 25.3% to 27.4% (Figure 1) and a relative increase (29.3–39.8%) in the class of reads longer than or equal to 38 nucleotides ( Additional file 1: Figure S4). With standard edit distances (−n 0.04), allowing two or three gaps in the alignment resulted in recovering hits with lower deamination levels, with G → A misincorporation levels of 12.5–12.7% at sequencing starts compared to 13.8% with one gap ( Additional file 1: Figure S2). Not surprisingly, indel rates increased, even to levels higher than the manufacturer’s specifications, suggesting that the latter could be slightly under-estimated or that misalignments were more often solved by introducing gaps than by mismatches, despite higher alignment penalty scores. Interestingly, a substantially higher fraction of simulated reads could be successfully mapped against the right location of the horse genome when allowing for a maximum number of two gap opens (from 57.5–61.7% to 67.5–75.7% of the reads simulated; Additional file 1: Table S1), suggesting the overall adequacy of this approach for mapping Helicos reads.

We further evaluated how much different gap open and extension penalties could influence mapping outcomes for Helicos tSMS reads, while still allowing a maximum of two gap opens. The performance and runtime of BWA were therefore examined for combinations of penalties ranging from four to eleven (Figure 1: Table 1). Decreasing gap open penalties was detrimental while gap extension penalties were found to have almost no influence on the performance of BWA mapping. In addition, all combinations of parameters explored were found to result in similar runtime cost. Consequently, default penalty values were found to perform better than most of the other combinations tested and were kept for further experiments.

**Table 1:** Exploring BWA performance and runtime with different combinations of penalties for opening and extending gaps on Helicos tSMS reads

		**Hits**							**Runtime**				
**Gap Extend Penalty**	**11**	9.9%	10.2%	22.9%	22.7%	25.5%	**Gap Extend Penalty**	**11**	1.7x	1.5x	1.6x	1.6x	1.6x
	**9**	9.9%	10.2%	22.9%	22.7%	25.5%		**9**	1.8x	1.6x	1.6x	1.6x	1.6x
	**7**	9.9%	10.2%	22.9%	22.7%	25.6%		**7**	1.7x	1.7x	1.6x	1.7x	1.7x
	**5**	9.7%	10.0%	22.6%	22.5%	25.3%		**5**	1.6x	1.7x	1.7x	1.7x	1.7x
	**4**	9.7%	9.9%	22.6%	22.5%	25.3%		**4**	1.6x	1.6x	1.8x	1.7x	1.6x
		**4**	**5**	**7**	**9**	**11**			**4**	**5**	**7**	**9**	**11**
		**Gap Open Penalty**							**Gap Open Penalty**				

Sequencing reads recovered from the sample showing infinite radiocarbon date were aligned using different combinations of penalties for opening (−O) and extending (−E) gaps using the BWA aligner with seed disabled (−l 1024) and allowing a maximum number of two indels (−o 2) possibly located at read ends (−i 0). Shifts in the percentage of high-quality hits recovered and total runtime are reported relative to the values observed using BWA default parameters, with the result for default gap penalties shown in green. Reads were considered of high-quality when mapping uniquely to the EquCab2 genome but not against the human genome (assembly hg19) and showing mapping qualities of at least 25.

### Increasing tolerance for higher edit distances

**Figure 2:**
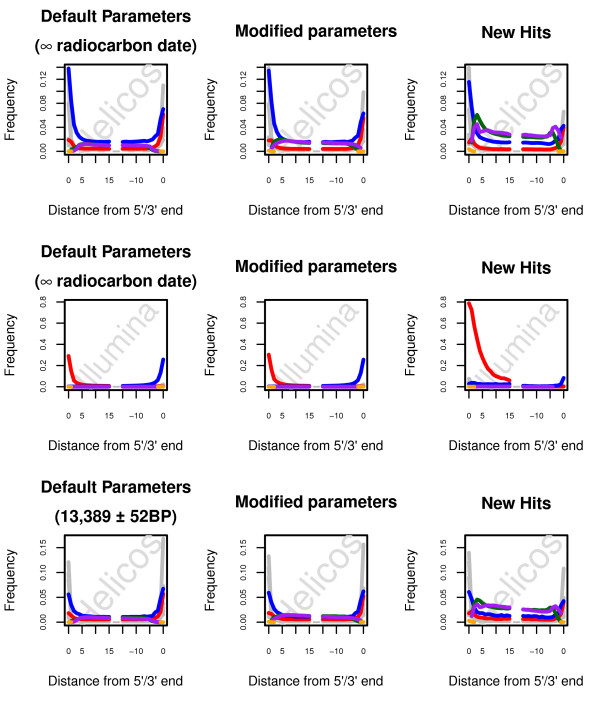
**Nucleotide misincorporation patterns observed with standard and optimized BWA mapping parameters.** Nucleotide misincorporation patterns observed when using the reads recovered from default or optimized BWA parameters are shown on the left and in the middle columns respectively. Nucleotide misincorporation patterns observed on the fraction of high-quality hits identified only with the optimized set of parameters are shown on the right. For both Illumina and Helicos sequencing data, the seed was disabled in the optimized set of mapping parameters (−l 1024). For Helicos tSMS reads, we further increased the maximum number of gap opens to 2 (−o 2) as well as the edit distance (−n 0.03) and allowed for indels at read termini (−i 0). Red: C → T. Blue: G → A. Pink: Insertions. Green: Deletions. Orange: Clipped bases. Grey: Other misincorporations.

For standard BWA settings, the maximum edit distance allowed in a given alignment is controlled dynamically according to read length. In the size range relevant in our case study (25–76 nucleotides), three mismatches are accepted at maximum for reads shorter than 38 nucleotides, four for reads shorter than 64 nucleotides, and five otherwise. These thresholds may be manipulated using the -n option, with a default value of 0.04. From default mapping parameters, the cumulative frequency of GC → AT misincorporations located in the five nucleotides at read termini could be estimated at approximately 32.1% and 55.9% using Helicos and Illumina reads respectively. This rate is even higher if determined over all positions (Figure 2). This suggests that *post-mortem* deaminations at cytosine residues contribute massively to the edit distance of alignments, limiting our ability to identify the fraction of endogenous reads that are most divergent. Increasing our tolerance for higher edit distances might help with recovering this fraction, at the risk of accepting a larger proportion of reads of exogenous origin.

In order to evaluate the consequence of relaxing the threshold, BWA mapping was performed with the set of parameters validated above, and relaxing the –n option to 0.02 and 0.03 (Figure 1). In the size range relevant to our case study, the former corresponds to a maximum of three mismatches for reads of 29 nucleotides or shorter, four mismatches for reads of 51 nucleotides or shorter, and five mismatches otherwise; for the latter, three mismatches are accepted for reads of 34 nucleotides or shorter, four for reads of 58 nucleotides or shorter, and five mismatches otherwise. Interestingly, these parameters show only moderate extra gains in high-quality hits for Illumina reads (1.1%; Figure 1). This population of new hits exhibited nucleotide mismatch patterns characteristic of aDNA reads ( Additional file 1: Figure S1), suggesting most of those reads were genuine horse sequences. Their identification required, however, extensive runtime costs; consequently, the default parameter value was used for further mapping tests performed with Illumina sequencing reads.

For Helicos tSMS reads, the fraction of reads identified as high-quality hits was found to increase when allowing a higher edit distance, with extra-gains ranging from 29.1% to 37.6%, at a significant increase in runtime cost (4.6X-13.0X; Figure 1). In agreement with what observed on Illumina reads, the new population of reads identified exhibits a nucleotide misincorporation pattern indicative of cytosine deamination at 5’-overhanging ends, and a predominance of G → A miscoding lesions over the whole sequence length ( Additional file 1: Figure S2), suggesting that those could be of endogenous origin.

In order to further evaluate the overall quality of these reads, Helicos reads were mapped against the chicken reference genome ( Additional file 1: Figure S5). The most permissive edit distance (option -n 0.02) was found to yield more hits on the chicken genome, especially for read sizes of 29 nucleotides which corresponds to the smallest length tolerating three mismatches. This behavior was not observed for the intermediate edit distance (option –n 0.03), as >95% of Helicos reads that could be successfully aligned to the chicken genome consisted of reads shorter than 27 nucleotides (Figure 2; Additional file 1: Figure S5). In contrast, the fraction of new horse hits identified with this set of parameters consisted of reads of size longer than 34 nucleotides (Figure 2; Additional file 1: Figure S4), suggesting that the option –n 0.03 could be used to improve our ability to identify endogenous reads without inflating false-positive rates (contrary to what has been observed for the most permissive edit distance).

### Final recommendations for mapping aDNA reads

For Illumina reads, the amount of high-quality hits was found to be rather insensitive to the set of mapping parameters explored, with marginal gains ranging from 0.9% to 1.1% (Figure 1). Importantly, disabling the seed resulted in an extra 0.9% of high-quality hits and identified a population of reads showing nucleotide misincorporation patterns characteristic of *post-mortem* damage and no reduction in read size, as would have been observed for spurious alignments. Given that this only doubled runtime cost, we therefore recommend that aDNA reads generated with the Illumina sequencing technology should be systematically mapped following this procedure. Other sets of parameters explored have been found to improve our ability to identify endogenous sequence reads, however at extensive runtime costs, and therefore should be not be recommended on a systematic basis.

**Figure 3:**
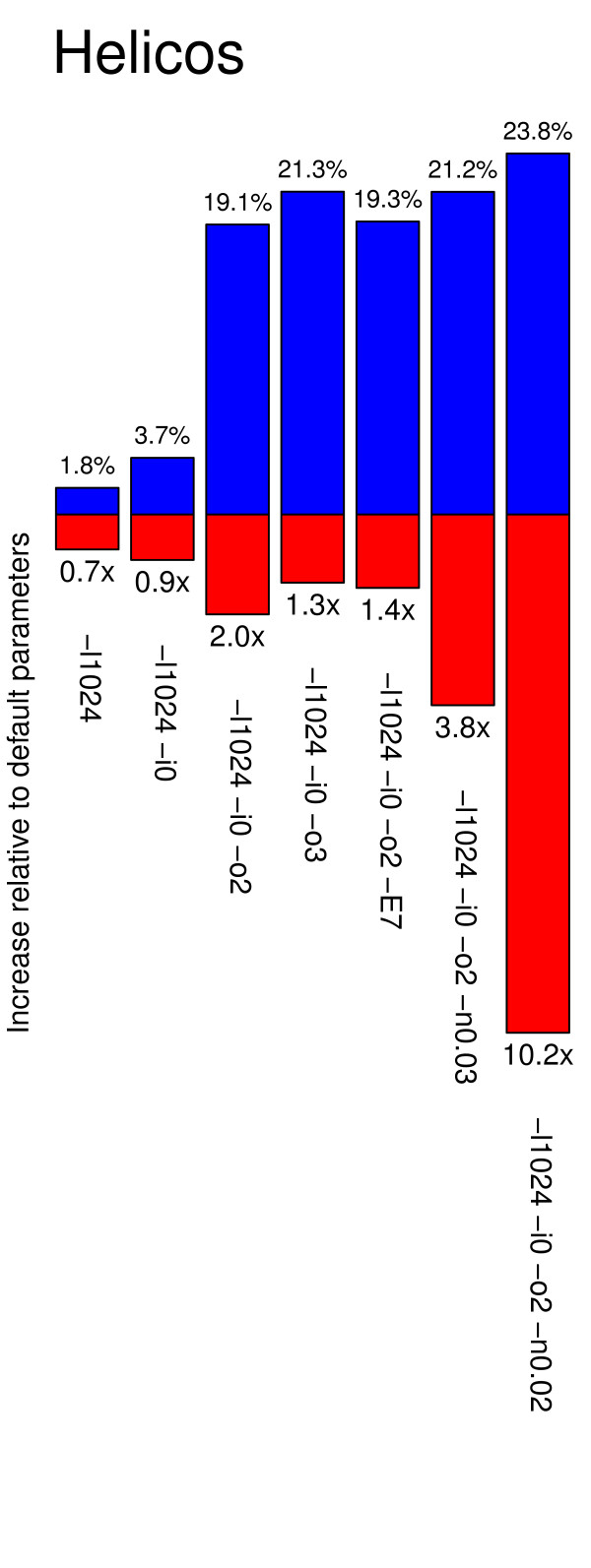
**Exploring the effects of different sets of mapping parameters on BWA performance and runtime.** Helicos sequencing reads recovered from the sample showing a finite radiocarbon date (13,389 ± 52BP) were aligned using different combinations of mapping parameters using the BWA aligner. Reads were considered of high-quality when mapping uniquely to the EquCab2 genome but not against the human genome (assembly hg19) and showing mapping qualities of at least 25. Performance and runtime are estimated with reference to the standard default parameters.

BWA parameters were found critical for mapping Helicos tSMS reads, with large increases in both the fraction of reads identified as high-quality and runtime costs (Figure 1). Of all settings tested, the best compromise was obtained by disabling the seed and tolerating up to two gap opens, even if located at read termini. Furthermore, the alignment edit distance could be relaxed slightly (option –n 0.03) with no apparent increase in spurious hits. However, tolerance for larger edit distances was found detrimental for the quality of the results and should be avoided. The same was observed for Helicos reads generated from a younger sample associated with a finite radio-carbon date, admittedly with more limited gains in high quality hits (21.2% extra gains of high quality hits with the suggested set of parameters; Figure 3). However, the suggested procedure was found to be computationally intensive, resulting in 3.8–4.6 fold increase in run time (Figures 1 and 3). The following experiments were performed according to the specific set of mapping parameters recommended for each sequencing platform.

### Trimming likely-damaged positions

Since nucleotide misincorporations are clustered at read termini, trimming mismatches resulting from *post-mortem* DNA damage at 5’-ends might further improve the chance of identifying endogenous DNA sequences. On the other hand, short reads are more likely to cause spurious alignments. In order to determine the read size that would minimize the occurrence of spurious alignments after trimming, Helicos reads, which are on average shorter than Illumina reads, were aligned against a genome distant to the horse, specifically the chicken. Even in absence of trimming and even though DNA was extracted from a horse fossil, reads shorter than 28 nucleotides were found to yield substantial amounts of high quality hits against the chicken genome ( Additional file 1: Figure S5).

**Figure 4:**
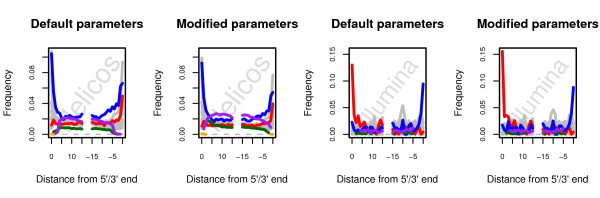
**Nucleotide misincorporation patterns observed with standard and optimized BWA mapping parameters for read mapping both the horse and the chicken genome.** Nucleotide misincorporation patterns observed for alignments against the horse genome (EquCab2), when using the reads recovered from default or optimized BWA parameters are shown on the left and on the right, respectively. For both Illumina and Helicos sequencing data, the seed was disabled in the optimized set of mapping parameters (−l 1024). For Helicos tSMS reads, we further increased the maximum number of gap opens to 2 (−o 2) as well as the edit distance (−n 0.03) and allowed for indels at read termini (−i 0). Reads were considered when mapping uniquely both to the EquCab2 and galGal3 genomes but not against the human genome (assembly hg19) and showing mapping qualities of at least 25. Red: C → T. Blue: G → A. Pink: Insertions. Green: Deletions. Orange: Clipped bases. Grey: Other misincorporations.

Taken at face value, the relatively high frequency of very short hits mapping against the chicken genome could suggest that a substantial fraction of the shortest horse hits are spurious. These hits could alternatively correspond to genomic regions that are ultra-conserved across vertebrates. Importantly, the large majority of high-quality alignments against the chicken genome (78.1–85.0%) did not show any match at all to the horse genome. A closer look at the fraction of reads mapping positively against both the chicken and horse genome revealed an increase in misincorporations related to cytosine *post-mortem* deamination (for Helicos, G → A mismatches at sequencing starts; C → T for Illumina), suggesting that at least a fraction of those consisted of genuine ancient horse reads mapping to genomic regions showing low sequence divergence between the chicken and the horse (Figure 4). Of note, for this fraction of hits, sequencing starts exhited 9.3% G → A misincorporations for Helicos and 15.6% for Illumina. This is lower than the rates observed when considering all horse high-quality hits (13.4% and 30.3%, respectively). This reduction is not unexpected as for short sizes, undamaged horse reads have higher chances of being identified than damaged horse reads and, hence, exhibit lower mismatch rates at 5’ ends. This phenomenon has been previously observed by Green and colleagues in the context of detecting derived alleles in short Neandertal DNA fragments [27]. Interestingly, we found an overall positive correlation between the levels of G → A mismatches and read length, both for Helicos reads (Pearson corre-lation coefficient = 0.46, *p*-value = 0.0078) and Illumina reads (Pearson correlation coefficient = 0.96, *p*-value < 2.2 x 10^−16^), emphasizing that mapping short reads is more likely to succeed for undamaged reads than for damaged reads. 

**Figure 5:**
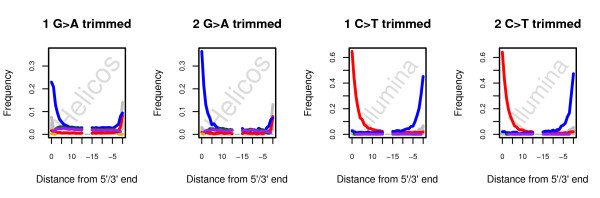
**Nucleotide misincorporation patterns observed after trimming the first or the first two bases of sequencing reads.** Nucleotide misincorporation patterns observed when using the reads recovered after trimming the first base (left) or the two first bases (right) of the sequencing reads, provided that in absence of trimming one or two successive nucleotide misincorporations would have been observed as a result of cytosine *post-mortem* deamination (see Methods). Red: C → T. Blue: G → A. Pink: Insertions. Green: Deletions. Orange: Clipped bases. Grey: Other misincorporations.

That a substantial number of high-quality hits were identified against the chicken genome for reads shorter than 28 nucleotides suggests that putative spurious hits would mostly consist of reads of similar or shorter sizes. Consequently, reads that did not previously align against the horse genome were filtered for sizes greater than or equal to 30 before being trimmed of one or two bases from the 5'-end and aligned against the horse and human genomes. Resulting hits were further binned based on whether or not the trimmed base would have caused C → T (Illumina) or G → A (Helicos) mismatches against the reference, and nucleotide misincorporation patterns were plotted (Figure 5). As a control, the procedure was carried out while selecting for non-damage-derived mismatches ( Additional file 1: Figure S8–S11).

For Illumina reads, trimming of a single C → T mismatch at 5’-ends yielded a 0.53% gain in high-quality-hits mapping against the horse reference ( Additional file 1: Table S2); additionally, the pattern of nucleotide misincorporation for the new hits is compatible with an ancient origin, showing the characteristic elevation of C → T mismatches at 5’-termini, up to levels that even exceeds the rate observed for the overall population of Illumina hits (64.8% in contrast to the 30.3% observed in Figure 2), paralleled by an increase in G → A mismatches at 3’-ends (Figure 5). Likewise, the trimming of two consecutive C → T mismatches resulted in similar findings, with an additional 0.64% extra reads of horse origin ( Additional file 1: Table S2).

In contrast, trimming of any other class of mismatch resulted in negligible gains ranging from 0.01–0.06%. Those reads could represent a fraction of the ancient horse genome that exhibits substantial polymorphisms to the ancient horse genome. However, the mismatch patterns observed after trimming most other types of mismatches was not found to show the expected signature of *post-mortem* cytosine deamination ( Additional file 1: Figure S10–S11), suggesting that the background increase in high-quality horse reads identified after trimming mismatches other than C → T mostly consists of modern DNA and/or spurious alignments.

A similar procedure was performed on Helicos tSMS reads, with more ambiguous results. Similar to Illumina reads, gains (0.9–1.07%) in high-quality horse reads were obtained after trimming one or two G → A mismatches at the very 5’ end, suggesting that the presence of *post-mortem* cytosine deamination prevented successful mapping before trimming (Figure 5; Additional file 1: Figure S8–S9; Additional file 1: Table S2). Of note, the new population of hits identified exhibits substantial levels of cytosine deamination, as suggested by high overall levels of G → A mismatches at the 5’-ends (Figure 5; Additional file 1: Figure S8–S9). However, the trimming of other classes of mismatches also yielded substantial gains in high-quality hits ( Additional file 1: Table S2) that exhibit G → A mismatch patterns similar to those observed without trimming ( Additional file 1: Figure S8–S9). This was particularly true when trimming A → C, A → G, A → T, G → T and C → T mismatches at the first position. This may be partly explained by the early incorporation of virtual terminator nucleotides, during the fill-in / blocking step prior to sequencing [26]. This would lead to the resulting sequence being prefixed with a Thymine, as a base from the poly-Adenine tail would be sequenced as part of the template strand [28].

While the above demonstrates that some additional aDNA fragments may be recovered by selectively trimming potentially damage-derived nucleotides, the gains were rather low (0.53–1.07%), in contrast to previous estimates based on simulated Helicos reads, which suggested a 5% loss of sequences due to *post-mortem* damage at the first position [15]. We believe that additional hits obtained through the manipulation of BWA parameters were drawn from the pool of sequences that could not be aligned due to the presence of *post-mortem* damage, reducing the effect of this operation. Given the relatively small gains and the additional complexity required for setting-up the analytical pipeline, we would generally refrain from recommending such a trimming approach.

### Filtering human contamination

**Figure 6:**
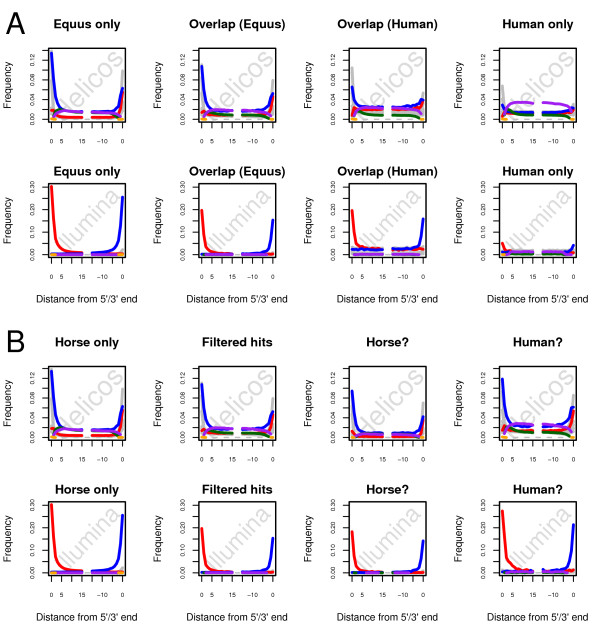
**Nucleotide misincorporation patterns observed following different filtering procedures for human sequences.** Helicos and Illumina sequencing reads recovered from the sample showing infinite radiocarbon date were aligned using different combinations of mapping parameters using the BWA aligner. Reads were considered of high-quality when mapping uniquely to the equCab2 genome but not against the human genome (assembly hg19) and showing mapping qualities of at least 25. In a first mapping procedure (**Panel A**), reads were considered of high-quality when mapping uniquely to the EquCab2 genome but not against the human genome (assembly hg19). In a second mapping procedure (**Panel B**), reads were considered of high-quality when mapping uniquely to the EquCab2 genome as long as no hit was observed against the human genome (assembly hg19) or as long as the edit distance to the horse genome was lower than the edit distance to the human genome. High-quality reads presented minimal mapping qualities of 25. Nucleotide misincorporation patterns were plotted following mapping with the optimized set of BWA parameters for different subsets of reads. **Panel A:** Alignments against the horse reference genome, excluding any read that also map against the human reference genome (first column); alignments against the horse reference genome, for reads that also map against the human reference genome (second column); alignments against the human reference genome, for reads that also map against the horse reference genome (third column); alignments against the human reference genome, excluding any read that also maps against the horse reference genome (last column). **Panel B:** reads showing hits to the horse reference genome only (first column); reads showing hits to the horse and the human reference genomes, and that were filtered in the filtering procedure presented on Panel A (second column); reads showing hits to the horse and the human reference genomes but a lower edit distance to the horse genome (third column); reads showing hits to the horse and the human reference genomes but a lower or equal edit distance to the human genome (last column). Red: C → T. Blue: G → A. Pink: Insertions. Green: Deletions. Orange: Clipped bases. Grey: Other misincorporations.

As presented above, hits against the *E. caballus* genome were aligned against the human genome, and high-quality hits were filtered as contamination. This accounts for 7.6% and 1.5% of the horse hits, for Helicos and Illumina data respectively. As human contamination appears to represent only a small subset of the total data [15], a high rate of false positives is expected, especially for ultra-conserved genomic regions. This is further attested by the mismatch patterns of hits mapping to both genomes, which show typical signatures of *post-mortem* cytosine deamination both for Illumina and Helicos reads, suggesting that reads filtered according to this procedure largely consist of ancient horse DNA (Figure 6). Consequently, additional authentic aDNA horse fragments might be recovered by improving the specificity of the filtering of putative human contamination. 

We examined the benefit of a commonly used strategy that has already been applied for filtering environmental contamination from Neandertal NGS reads [29] but also in many other contexts (e.g. expression profiling) [30]. This method is based on the edit-distance of potential contamination to different target genomes (here, of human and horse). Following a best hit criterion, reads of a likely human origin were identified as those with a higher edit-distance to the horse genome than to the human genome. Using simulation, we could show that a substantially higher fraction of horse high-quality hits (4.5%–6.7%) could be identified (data not shown).

When applied to the real sequencing data, the modified filtering criteria led to the recovery of 40.5% of hits previously filtered as human for Helicos, and 85.7% for Illumina (data not shown). The smaller fraction of previously filtered hits recovered for the Helicos platform may be explained as a consequence of the shorter read lengths, which increases the chance alignment of a horse read to a homologous region in the human genome. To examine the adequacy of this new filtering approach, nucleotide misincorporation patterns were plotted for reads mapping only to the horse genome, and for reads mapping to both the horse and human genomes (Figure 6). In the latter case, nucleotide misincorporation patterns were further examined for reads showing a lower edit distance to the horse, or equal or lower edit distances to the human genome (Figure 6). Interestingly, reads mapping against both the horse and human genome showed nucleotide misincorporation patterns indicative of *post-mortem* cytosine deamination, regardless of the edit distance to the horse genome (Figure 6). As reads mapping uniquely against the human genome did not exhibit any trace of *post-mortem* cytosine deamination (Figure 6), this suggests that a substantial fraction of the reads filtered as putatively originating from a human source actually consisted of genuine horse reads, that are still filtered from our analytical pipeline. Interestingly, G → A (Helicos) and C → T (Illumina) misincorporation rates at 5’-ends were found substantially higher for the fraction of reads showing a lower edit distance to the horse genome. This most likely results from the edit-distance, as we favor those hits that show fewer mismatches against the horse genome, including hits with less damage. While our filtering approach discarded a substantial fraction of potentially endogenous hits (59.5% and 14.3% for Helicos and Illumina, respectively), we suggest that the above method is still more valuable than the first approach where all hits to the horse genome that also mapped to the human genome were eliminated. This approach can be used to reduce the fraction of endogenous DNA sequences that would have been filtered otherwise due to high proximity to human genomic sequences.

### Divergence estimates

**Figure 7:**
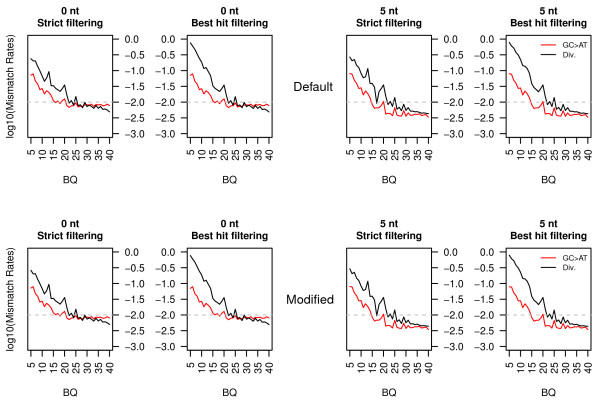
**Divergence estimates based on different mapping and filtering procedures.** Illumina reads recovered from the sample showing infinite radiocarbon date were aligned using the default (top) or the recommended modified (bottom) combination of mapping parameters using the BWA aligner. High-quality hits were further filtered according to a strict (no hit on the human genome) or a best hit criterion (horse high-quality reads are discarded if showing better alternative hit on the human genome). Divergence to the modern reference genome and GC → AT misincorporation rates were calculated and reported with black and red lines as a function of base quality scores. Reads were either considered full length (0 nt) or masked for 5 nucleotides at both ends (5 nt). Further trimming (10 nucleotides) was performed and showed similar results (data not shown). BQ: Base-Quality.

The set of alignment parameters has shown a significant impact on the population of sequencing reads identified as endogenous by BWA. Reads with extremely high nucleotide misincorporation rates at the 5’-ends are disregarded with default parameters but are recovered when mapping is performed with no seed (Figure 1) or after the first (or the two first) nucleotides are trimmed (Figure 5). Additionally, we have shown that excluding reads based on their mapping onto the human genome could result in a substantial loss of potentially endogenous hits ( Additional file 1: Table S3). We further investigated how these different approaches could affect divergence estimates between the ancient sample and the modern reference. In addition to true biological mutations, a number of other phenomena contribute to the total number of differences observed between sequencing reads and the reference: sequencing errors, *post-mortem* damage (and related misincorporations) and local misalignments. Base qualities and GC → AT misincorporations could be used as proxies for sequencing errors and *post-mortem* damage, respectively. However, not all GC → AT substitutions originate from *post-mortem* cytosine deamination and a number equivalent to the reciprocal class of substitution (namely, AT → GC) would be expected as a result of the process of lineage divergence. Hence, a simple measure of divergence could be estimated by summing over sites showing high base quality scores all classes of transversions, indels and by doubling the number of AT → GC transitions. This estimate is reported with a black line on Figure 7 as a function of base quality scores (BQ) for Illumina reads (Helicos reads do not include base quality scores and were therefore disregarded in this analysis). Not unexpectedly, divergence estimates increased drastically with low BQ as a result of sequencing errors. For BQ ≤ 25, sequencing errors even exceeded nucleotide misincorporations resulting from *post-mortem* cytosine deamination (GC → AT; Figure 7, red line). High base quality scores (BQ ≥ 35), however, provide divergence estimates ranging 0.489%–0.730%. Of note, neither read trimming nor changing the mapping procedure to the new set of modified parameters seemed to affect divergence, with estimates ranging from 0.426% to 0.725% for BQ ≥ 35 (Figure 7). Read trimming, however, reduced (but not eliminated) GC → AT misincorporation rates in agreement to the higher cytosine deamination rates observed at overhanging ends in ancient DNA fragments [19]. 

The reference used for mapping could also represent another potential source of bias for divergence estimates as reads showing low, if any, divergence to the reference exhibit higher mapability success. Yet, the different sets of alignment parameters investigated here do not have similar tolerance for highly divergent reads. Therefore, we investigated whether standard and the suggested modified alignment parameters will provide similar divergence estimates between the ancient and modern horse. In absence of a complete genome from an ancient horse and from a non-caballine equid, we decided to restrict the analysis to the mitochondrial genome that is fully sequenced in donkeys (Accession number: NC_001788). Interestingly, although more hits were recovered using our modified parameters, no significant difference in the relative numbers of reads mapping the horse or the donkey mitochondrial genome was detected when using the standard or the different sets of parameters recommended here (chi-square test, p-value = 0.808 and 0.854, for Illumina and Helicos platforms, respectively). Thus, regardless of which alignment parameter was used, similar divergence estimates were recovered between the ancient sample and the horse mitochondrial genome reference (BQ ≥ 35: 1.240–1.288%) when using the horse mitochondrial as a reference for mapping (Accession number: NC_001640). Lower estimates were found when using the donkey mitogenome for mapping and realigning the reads identified to the horse (BQ ≥ 35: 0.829–0.951%). This illustrates that mapping against distant genome references leads to a significant under-estimation of divergence estimates, likely due to the fact that most divergent reads are missed. It follows that it is unlikely that the nuclear divergence estimate provided above was underestimated, given that the mitochondrial divergence was found to be 2- to 3-fold higher, suggesting the ability of our mapping parameters to identify most divergent reads. Therefore, as it provides unbiased estimates of sample divergence while increasing sequence coverage, we believe that the approach recommended here would improve the sensitivity of the mapping procedure in other situations where ancient reads show similar levels of *post-mortem* damage and could accommodate divergence estimates up to at least 1.2%.

## Conclusions

In this study, we have demonstrated significant improvements in the identification of aDNA reads deriving from shotgun sequencing data, by optimizing the computational procedure used to identify endogenous DNA. We have shown that Illumina and Helicos sequencing data recovered from aDNA extracts could not be aligned to modern reference genome sequences with the same efficiency unless mapping parameters are optimized both for the specific types of errors generated by these platforms and for *post-mortem* DNA damage. For Helicos tSMS reads, we have shown that simple modifications to the behavior of the semi-global aligner can yield a 1.6%–29.1% increase in the amount of endogenous sequences identified. Similar modifications yield a smaller increase for Illumina sequencing data, most likely due to the longer reads and higher information content present in the sequences generated on that platform. Additionally, we have shown that selective trimming of nucleotide misincorporations that result from *post-mortem* damage at overhanging ends of template strands could only slightly improve our abi-lity to identify endogenous sequences for Helicos tSMS sequencing data on samples showing high levels of cytosine deamination. This procedure was found to have only moderate effects for Illumina reads. We have shown that additional gains could be achieved by filtering putative human contamination according to the edit distance of the alignment.

The fact that the specific nature of aDNA together with the specificities of sequencing platforms must be taken into account when attempting to identify endogenous aDNA fragments has important implications for future aDNA research, as our findings make it possible to recover a greater amount of endogenous DNA sequences with no need for extra sequencing runs and further destruction of fossil samples. Together with recent developments in molecular methods for more specific extraction, capturing and/or sequencing of aDNA molecules [9,15,16,21,31,32], the series of computational approaches presented in this study offer an additional technique for maximizing the recovery of genome-wide sequence information from ancient individuals and extinct species in a more efficient way. Our findings pleads for the development of mapping software dedicated to the specificities of aDNA reads, combining high rates of particular classes of nucleotide misincorporations at read termini together with short read sizes. For now, this development is still in its infancy, with programs such as MIA [33], sesam [7] and ANFO [19] using nucleotide misincorporation patterns resulting from *post-mortem* damage in their scoring schemes, but most algorithms have limited use due to excessive runtime costs. We are convinced that such developments will contribute tremendously to the success of large-scale paleogenomic projects as it will improve our ability to identify the genuine fraction of reads originating from ancient samples in which contamination is inevitable.

## Methods

### Sequence alignment

The DNA sequences used in this manuscript have already been reported in [15] and [16] and are available on the NCBI Sequence Read Archive (SRA Accession Numbers: SRP005902 and SRA0458620). They consist of a total of 12 Helicos tSMS channels (157,298,986 sequences) and 7 Illumina GAIIx lanes (172,991,377 sequences) generated from a series of DNA extracts of a horse bone sample associated with infinite radiocarbon date (OxA-23933 >50,300BP), and of 12 Helicos tSMS channels (7,976,053 sequences) from different DNA extracts of a horse bone sample radiocarbon-dated at 13,389 ± 52BP (UBA-16479). Raw Illumina sequencing reads were pre-processed following the procedure described in [15] and trimmed for residual adaptor sequences and regions starting or ending with minimal PHRED quality-scores; additionally, sequencing starts were filtered for undetermined bases and/or minimal quality scores. Additionally, reads shorter than 25 nucleotides were discarded to reduce the chance of spurious hits against the horse reference genome.

Putative horse DNA fragments were identified by mapping sequencing reads against the 2009 *Equus caballus* draft assembly (EquCab2) [34] using BWA [18]. Likewise, reads resulting from human contamination were identified using the hg19 human reference genome, including assembled chromosomes, unplaced and unlocalized sequences, alternative chromosome 6 haplotype assemblies, and using the revised Cambridge Reference Sequence for the mitochondrial genome. To estimate the rate of spurious alignments, reads were aligned against the genome of the modern chicken (*Gallus gallus*) using the May 2006, galGal3 assembly available at UCSC ( http://hgdownload.cse.ucsc.edu/downloads.html#chicken).

Initial alignments were carried out using BWA v0.5.9, with default “aln” and “samse” parameters, except that multiple threads (6) were used. This procedure corresponds to a maximum edit distance of 2 in the 32 nucleotide-long seed region, at most one gap opened and no indels within the five nucleotides located at both sequence termini. By default, mismatch, gap open and gap extension penalties were set to 3, 11 and 4, respectively. We further explored different combinations of alignment parameters, including: no seeding (−l 1024); tolerating indels at both sequence termini (−i 0); opening a maximum number of 2 (−o 2) or 3 (−o 3) indels; changing the range of penalties for opening (−O) and extending (−E) gaps to 4, 5, 7, 9, and 11; considering the overall number of mismatches tolerated in the alignment by setting the expected fraction of misalignments to 0.03 and 0.02 (−n). Following these tests, an optimized vector of parameters was selected for both Illumina and Helicos reads; all the analyses described below were performed using these optimized sets of parameters, that consist of –l 1024 for Illumina data and –l 1024 –i 0 –o 2 -n 0.03 for Helicos data, respectively disabling the seed region (by setting it to a value longer than the longest query sequence), and additionally for Helicos allowing indels along the entire length of the alignment, including termini, allowing a maximum number of 2 gaps during the alignment, and increasing the maximum number of mismatches allowed in a length-dependent manner.

Following alignment, Illumina lanes were merged into a single dataset, and PCR duplicates were flagged using MarkDuplicates from the Picard suite of tools ( http://picard.sourceforge.net). For all BWA alignments, the term “high-quality hits” refers to uniquely mapped reads, as specified by the XT tag, having a mapping quality of at least 25, and no suboptimal alternative hits as specified by the X1 tag. Unless otherwise specified (e.g. when filtering contamination, see below), high-quality hits do not include Illumina hits that have been flagged as PCR duplicates. When reported, runtime is measured using the shell “time” command, and refers to the total time spent in user space for running an alignment.

### Filtering contamination

Unless otherwise specified (section ‘Filtering human contamination’), any sequence that yielded a high-quality hit (see above) against both the horse genome and against the human genome was excluded in order to filter out possible contamination prior to analysis. In the case of Illumina lanes, all human hits were considered, even when exhibiting alternative hits (paralogs) as a conservative approach. A second filtering option was examined, namely filtering putative horse hits as long as they exhibited a greater or equal edit-distance to EquCab2 than to hg19.

### Mismatch rate estimates

Nucleotide misincorporation rates were estimated following the methodology of the mapDamage package developed by Ginolhac and colleagues [20]. Position specific error rates were estimated by dividing the number of mismatches observed at each position across the alignments by the total number of nucleotides observed in the reference genome at the corresponding positions. The rates of insertions, deletions and (soft) clipped bases were calculated by dividing the number of nucleotides respectively inserted, deleted or clipped at a given position by the total read depth at that position. In contrast to the parameters used by default in the mapDamage package, 15 nucleotides (instead of 20) were considered at read termini.

### Trimming of damaged nucleotides

Reads that did not qualify as high-quality in the procedure presented above were further trimmed for one or two bases from the 5’-end. The trimming was not extended further into the reads as the two first positions, which account for most of the cytosine deaminations (Figure 2). Only reads with a minimal sequence length of 30 nucleotides before trimming were considered in order to preserve sufficient sequence information after trimming. The trimming procedure was carried out both blind, and considering the nature of the bases to be trimmed as DNA damage is most likely to generate C→T and G→A mismatches at 5’-ends of Illumina and Helicos reads, respectively [15,19]. Trimmed datasets were generated by trimming all reads of each lane / channel, and aligning these against the EquCab2 and hg19 genomes. Using information from the untrimmed reads, and from the reference genomes, the mismatches that would have occurred if the bases were not trimmed could be determined. Filtering of contamination was performed as described in the previous section, with the addition that alignments were filtered if either the untrimmed or trimmed sequence matched against the human genome. This conservative approach was carried out in order to limit chance alignments, as reducing the overall length of query sequences is expected to inflate the rates of spurious alignments.

### Simulation of helicos and random reads

Read lengths for simulated reads were selected based on the observed distribution of hits unique to the human or horse genome, along with the distribution of reads per chromosome. Start-sites on each chromosome (both strands merged into one sequence) were selected according to the distribution observed for the aforementioned hits, using a PWM based on positions −5:5 from the 5' termini of observed hits to score each potential position in the genome prior to sampling. For each read, bases were read from the selected start-site until the desired length was reached, using fixed rates of insertions (1.5%) and deletions (3%), adopting rates reported by Helicos BioSciences, and range of several possible rates of substitution errors (0, 0.5, 1.0, and 1.5%), based on the underlying distribution of nucleotides to select the substituting nucleotide. Reads containing N's, or overlapping both strand sequences, were discarded.

Random sequences were generated using the length distribution observed for all reads longer than 24 bp, and using the observed distribution of nucleotides to select the base at each position.

## Abbreviations

aDNA: ancient DNA; BWA: Burrows-Wheeler Aligner; tSMS: true Single Molecule DNA Sequencing.

## Competing interests

John F. Thompson was an employee of Helicos Biosciences. He now works at NABsys Inc.

## Authors’ contributions

MS carried out sequence analyses. MS, AG, SL, AK and LO designed the study. KAR, EW, AK and LO provided reagents, methods and tools. MS and LO wrote the manuscript. All authors read and approved the final manuscript.

## Supplementary Material

Additional file 1 Supplementary information for “Improving ancient DNA read mapping against modern reference genomes”. Click here for file

## References

[B1] Willerslev E Hansen AJ Rønn R Brand TB Barnes I Wiuf C Gilichinsky D Mitchell D Cooper A Long-term persistence of bacterial DNA Curr Biol 2004 14 R9 R10 10.1016/j.cub.2003.12.012 14711425

[B2] Gilbert MTP Bandelt H-J Hofreiter M Barnes I Assessing ancient DNA studies Trends Ecol Evol 2005 20 541 544 10.1016/j.tree.2005.07.005 16701432

[B3] Willerslev E Cappellini E Boomsma W Nielsen R Hebsgaard MB Brand TB Hofreiter M Bunce M Poinar HN Dahl-Jensen D Johnsen S Steffensen JP Bennike O Schwenninger J-L Nathan R Armitage S de Hoog C-J Alfimov V Christl M Beer J Muscheler R Barker J Sharp M Penkman KEH Haile J Taberlet P Gilbert MTP Casoli A Campani E Collins MJ Ancient biomolecules from deep ice cores reveal a forested southern Greenland Science 2007 317 111 114 10.1126/science.1141758 17615355 PMC2694912

[B4] Stiller M Baryshnikov G Bocherens H AG d’Anglade null Hilpert B Münzel SC Pinhasi R Rabeder G Rosendahl W Trinkaus E Hofreiter M Knapp M Withering away--25,000 years of genetic decline preceded cave bear extinction Mol Biol Evol 2010 27 975 978 10.1093/molbev/msq083 20335279

[B5] Lorenzen ED Nogués-Bravo D Orlando L Weinstock J Binladen J Marske KA Ugan A Borregaard MK Gilbert MTP Nielsen R Ho SYW Goebel T Graf KE Byers D Stenderup JT Rasmussen M Campos PF Leonard JA Koepfli K-P Froese D Zazula G Stafford TW Aaris-Sørensen K Batra P Haywood AM Singarayer JS Valdes PJ Boeskorov G Burns JA Davydov SP Haile J Jenkins DL Kosintsev P Kuznetsova T Lai X Martin LD McDonald HG Mol D Meldgaard M Munch K Stephan E Sablin M Sommer RS Sipko T Scott E Suchard MA Tikhonov A Willerslev R Wayne RK Cooper A Hofreiter M Sher A Shapiro B Rahbek C Willerslev E Species-specific responses of Late Quaternary megafauna to climate and humans Nature 2011 479 359 364 10.1038/nature10574 22048313 PMC4070744

[B6] Gilbert MTP Kivisild T Grønnow B Andersen PK Metspalu E Reidla M Tamm E Axelsson E Götherström A Campos PF Rasmussen M Metspalu M Higham TFG Schwenninger J-L Nathan R Hoog C-JD Koch A Møller LN Andreasen C Meldgaard M Villems R Bendixen C Willerslev E Paleo-Eskimo mtDNA genome reveals matrilineal discontinuity in Greenland Science 2008 320 1787 1789 10.1126/science.1159750 18511654

[B7] Rasmussen M Li Y Lindgreen S Pedersen JS Albrechtsen A Moltke I Metspalu M Metspalu E Kivisild T Gupta R Bertalan M Nielsen K Gilbert MTP Wang Y Raghavan M Campos PF Kamp HM Wilson AS Gledhill A Tridico S Bunce M Lorenzen ED Binladen J Guo X Zhao J Zhang X Zhang H Li Z Chen M Orlando L Kristiansen K Bak M Tommerup N Bendixen C Pierre TL Grønnow B Meldgaard M Andreasen C Fedorova SA Osipova LP Higham TFG Ramsey CB Hansen TVO Nielsen FC Crawford MH Brunak S Sicheritz-Pontén T Villems R Nielsen R Krogh A Wang J Willerslev E Ancient human genome sequence of an extinct Palaeo-Eskimo Nature 2010 463 757 762 10.1038/nature08835 20148029 PMC3951495

[B8] Rasmussen M Guo X Wang Y Lohmueller KE Rasmussen S Albrechtsen A Skotte L Lindgreen S Metspalu M Jombart T Kivisild T Zhai W Eriksson A Manica A Orlando L Vega FMDL Tridico S Metspalu E Nielsen K Ávila-Arcos MC Moreno-Mayar JV Muller C Dortch J Gilbert MTP Lund O Wesolowska A Karmin M Weinert LA Wang B Li J Tai S Xiao F Hanihara T van Driem G Jha AR Ricaut F-X de Knijff P Migliano AB Romero IG Kristiansen K Lambert DM Brunak S Forster P Brinkmann B Nehlich O Bunce M Richards M Gupta R Bustamante CD Krogh A Foley RA Lahr MM Balloux F Sicheritz-Pontén T Villems R Nielsen R Wang J Willerslev E An Aboriginal Australian genome reveals separate human dispersals into Asia Science 2011 334 94 98 10.1126/science.1211177 21940856 PMC3991479

[B9] Green RE Krause J Briggs AW Maricic T Stenzel U Kircher M Patterson N Li H Zhai W Fritz MH-Y Hansen NF Durand EY Malaspinas A-S Jensen JD Marques-Bonet T Alkan C Prüfer K Meyer M Burbano HA Good JM Schultz R Aximu-Petri A Butthof A Höber B Höffner B Siegemund M Weihmann A Nusbaum C Lander ES Russ C Novod N Affourtit J Egholm M Verna C Rudan P Brajkovic D Kucan Z Gusic I Doronichev VB Golovanova LV Lalueza-Fox C de la Rasilla M Fortea J Rosas A Schmitz RW Johnson PLF Eichler EE Falush D Birney E Mullikin JC Slatkin M Nielsen R Kelso J Lachmann M Reich D Pääbo S A draft sequence of the Neandertal genome Science 2010 328 710 722 10.1126/science.1188021 20448178 PMC5100745

[B10] Krause J Fu Q Good JM Viola B Shunkov MV Derevianko AP Pääbo S The complete mitochondrial DNA genome of an unknown hominin from southern Siberia Nature 2010 464 894 897 10.1038/nature08976 20336068 PMC10152974

[B11] Reich D Green RE Kircher M Krause J Patterson N Durand EY Viola B Briggs AW Stenzel U Johnson PLF Maricic T Good JM Marques-Bonet T Alkan C Fu Q Mallick S Li H Meyer M Eichler EE Stoneking M Richards M Talamo S Shunkov MV Derevianko AP Hublin J-J Kelso J Slatkin M Pääbo S Genetic history of an archaic hominin group from Denisova Cave in Siberia Nature 2010 468 1053 1060 10.1038/nature09710 21179161 PMC4306417

[B12] Tumpey TM García-Sastre A Taubenberger JK Palese P Swayne DE Basler CF Pathogenicity and immunogenicity of influenza viruses with genes from the 1918 pandemic virus Proc Natl Acad Sci USA 2004 101 3166 3171 10.1073/pnas.0308391100 14963236 PMC365761

[B13] Miller W Drautz DI Ratan A Pusey B Qi J Lesk AM Tomsho LP Packard MD Zhao F Sher A Tikhonov A Raney B Patterson N Lindblad-Toh K Lander ES Knight JR Irzyk GP Fredrikson KM Harkins TT Sheridan S Pringle T Schuster SC Sequencing the nuclear genome of the extinct woolly mammoth Nature 2008 456 387 390 10.1038/nature07446 19020620

[B14] Green RE Krause J Ptak SE Briggs AW Ronan MT Simons JF Du L Egholm M Rothberg JM Paunovic M Pääbo S Analysis of one million base pairs of Neanderthal DNA Nature 2006 444 330 336 10.1038/nature05336 17108958

[B15] Orlando L Ginolhac A Raghavan M Vilstrup J Rasmussen M Magnussen K Steinmann K Kapranov P Thompson JF Zazula G Froese D Moltke I Shapiro B Hofreiter M Al-Rasheid KAS Gilbert MTP Willerslev E True single-molecule DNA sequencing of a Pleistocene horse bone Genome Res 2011 21 1705 1719 10.1101/gr.122747.111 21803858 PMC3202287

[B16] Ginolhac A Vilstrup J Stenderup J Rasmussen M Stiller M Shapiro B Zazula G Froese G Steinmann K Thompson JF Al-Rasheid K Gilbert MTP Willerslev E Orlando L Improving the performance of true single molecule sequencing for ancient DNA BMC Genomicsin press (MS# 8945021646177089) 10.1186/1471-2164-13-177 PMC3430569 22574620

[B17] Bowers J Mitchell J Beer E Buzby PR Causey M Efcavitch JW Jarosz M Krzymanska-Olejnik E Kung L Lipson D Lowman GM Marappan S McInerney P Platt A Roy A Siddiqi SM Steinmann K Thompson JF Virtual terminator nucleotides for next-generation DNA sequencing Nat Methods 2009 6 593 595 10.1038/nmeth.1354 19620973 PMC2719685

[B18] Li H Durbin R Fast and accurate short read alignment with Burrows-Wheeler transform Bioinformatics 2009 25 1754 1760 10.1093/bioinformatics/btp324 19451168 PMC2705234

[B19] Briggs AW Stenzel U Johnson PLF Green RE Kelso J Prüfer K Meyer M Krause J Ronan MT Lachmann M Pääbo S Patterns of damage in genomic DNA sequences from a Neandertal Proc Natl Acad Sci USA 2007 104 14616 14621 10.1073/pnas.0704665104 17715061 PMC1976210

[B20] Ginolhac A Rasmussen M Gilbert MTP Willerslev E Orlando L mapDamage: testing for damage patterns in ancient DNA sequences Bioinformatics 2011 27 2153 2155 10.1093/bioinformatics/btr347 21659319

[B21] Schuenemann VJ Bos K Dewitte S Schmedes S Jamieson J Mittnik A Forrest S Coombes BK Wood JW Earn DJD White W Krause J Poinar HN Targeted enrichment of ancient pathogens yielding the pPCP1 plasmid of Yersinia pestis from victims of the Black Death Proc Natl Acad Sci USA 2011 108 E746 E752 10.1073/pnas.1105107108 21876176 PMC3179067

[B22] Briggs AW Stenzel U Meyer M Krause J Kircher M Paabo S Removal of deaminated cytosines and detection of in vivo methylation in ancient DNA Nuc Acids Res 2010 38 e87 10.1093/nar/gkp1163 PMC2847228 20028723

[B23] Stiller M Green RE Ronan M Simons JF Du L He W Egholm M Rothberg JM Keates SG Keats SG Ovodov ND Antipina EE Baryshnikov GF Kuzmin YV Vasilevski AA Wuenschell GE Termini J Hofreiter M Jaenicke-Després V Pääbo S Patterns of nucleotide misincorporations during enzymatic amplification and direct large-scale sequencing of ancient DNA Proc Natl Acad Sci USA 2006 103 13578 13584 10.1073/pnas.0605327103 16938852 PMC1564221

[B24] Gilbert MTP Binladen J Miller W Wiuf C Willerslev E Poinar H Carlson JE Leebens-Mack JH Schuster SC Recharacterization of ancient DNA miscoding lesions: insights in the era of sequencing-by-synthesis Nucleic Acids Res 2007 35 1 10 16920744 10.1093/nar/gkl483 PMC1802572

[B25] Brotherton P Endicott P Sanchez JJ Beaumont M Barnett R Austin J Cooper A Novel high-resolution characterization of ancient DNA reveals C > U-type base modification events as the sole cause of post mortem miscoding lesions Nucleic Acids Res 2007 35 5717 5728 10.1093/nar/gkm588 17715147 PMC2034480

[B26] Hart C Lipson D Ozsolak F Raz T Steinmann K Thompson J Milos PM Single-molecule sequencing: sequence methods to enable accurate quantitation Methods Enzymol 2010 472 407 430 20580974 10.1016/S0076-6879(10)72002-4

[B27] Green RE Briggs AW Krause J Prüfer K Burbano HA Siebauer M Lachmann M Pääbo S The Neandertal genome and ancient DNA authenticity EMBO J 2009 28 2494 2502 10.1038/emboj.2009.222 19661919 PMC2725275

[B28] Thompson JF Steinmann KE Single molecule sequencing with a HeliScope genetic analysis system Curr Protoc Mol Biol 2010 Chapter 7:Unit7.10 10.1002/0471142727.mb0710s92 PMC2954431 20890904

[B29] Prüfer K Stenzel U Hofreiter M Pääbo S Kelso J Green RE Computational challenges in the analysis of ancient DNA Genome Biol 11 R47 20441577 10.1186/gb-2010-11-5-r47 PMC2898072

[B30] Creighton CJ Reid JG Gunaratne PH Expression profiling of microRNAs by deep sequencing Brief Bioinform 10 490 497 19332473 10.1093/bib/bbp019 PMC2733187

[B31] Maricic T Whitten M Pääbo S Multiplexed DNA sequence capture of mitochondrial genomes using PCR products PLoS One 2010 5 e14004 10.1371/journal.pone.0014004 21103372 PMC2982832

[B32] Bos KI Schuenemann VJ Golding GB Burbano HA Waglechner N Coombes BK McPhee JB Dewitte SN Meyer M Schmedes S Wood J Earn DJD Herring DA Bauer P Poinar HN Krause J A draft genome of Yersinia pestis from victims of the Black Death Nature 2011 478 506 510 10.1038/nature10549 21993626 PMC3690193

[B33] Briggs AW Good JM Green RE Krause J Maricic T Stenzel U Lalueza-Fox C Rudan P Brajkovic D Kucan Z Gusic I Schmitz R Doronichev VB Golovanova LV de la Rasilla M Fortea J Rosas A Pääbo S Targeted retrieval and analysis of five Neandertal mtDNA genomes Science 2009 325 318 321 10.1126/science.1174462 19608918

[B34] Wade CM Giulotto E Sigurdsson S Zoli M Gnerre S Imsland F Lear TL Adelson DL Bailey E Bellone RR Blöcker H Distl O Edgar RC Garber M Leeb T Mauceli E MacLeod JN Penedo MCT Raison JM Sharpe T Vogel J Andersson L Antczak DF Biagi T Binns MM Chowdhary BP Coleman SJ Valle GD Fryc S Guérin G Hasegawa T Hill EW Jurka J Kiialainen A Lindgren G Liu J Magnani E Mickelson JR Murray J Nergadze SG Onofrio R Pedroni S Piras MF Raudsepp T Rocchi M Røed KH Ryder OA Searle S Skow L Swinburne JE Syvänen AC Tozaki T Valberg SJ Vaudin M White JR Zody MC Platform BIGS Team BIWGA Lander ES Lindblad-Toh K Genome sequence, comparative analysis, and population genetics of the domestic horse Science 2009 326 865 867 10.1126/science.1178158 19892987 PMC3785132

