# Supplementary material for: Improving ancient DNA read mapping against modern reference genomes
Source: BMC Genomics. 2012 May 10;13:178. doi: 10.1186/1471-2164-13-178 (PMC3468387; doi:10.1186/1471-2164-13-178)
Supplement: Additional file 1 — Supplementary information for “Improving ancient DNA read mapping against modern reference genomes”. [file 1471-2164-13-178-S1.pdf]

Supplementary information for “Improving ancient DNA read mapping against modern reference genomes”

Mikkel Schubert, Aurelien Ginolhac, Stinus Lindgreen, John F. Thompson, Khaled A.S. AL-Rasheid, Eske Willerslev, Anders Krogh, and Ludovic Orlando.

Contents

Supplementary Figures **2**
Supplementary Figure 1 2
Supplementary Figure 2 4
Supplementary Figure 3 6
Supplementary Figure 4 7
Supplementary Figure 5 8
Supplementary Figure 6 9
Supplementary Figure 7 10
Supplementary Figure 8 12
Supplementary Figure 9 13
Supplementary Figure 10 14
Supplementary Figure 11 15

Supplementary Tables **16**
Supplementary table 1 16
Supplementary table 2 17
Supplementary table 3 18

Supplementary Figures

Supplementary Figure 1

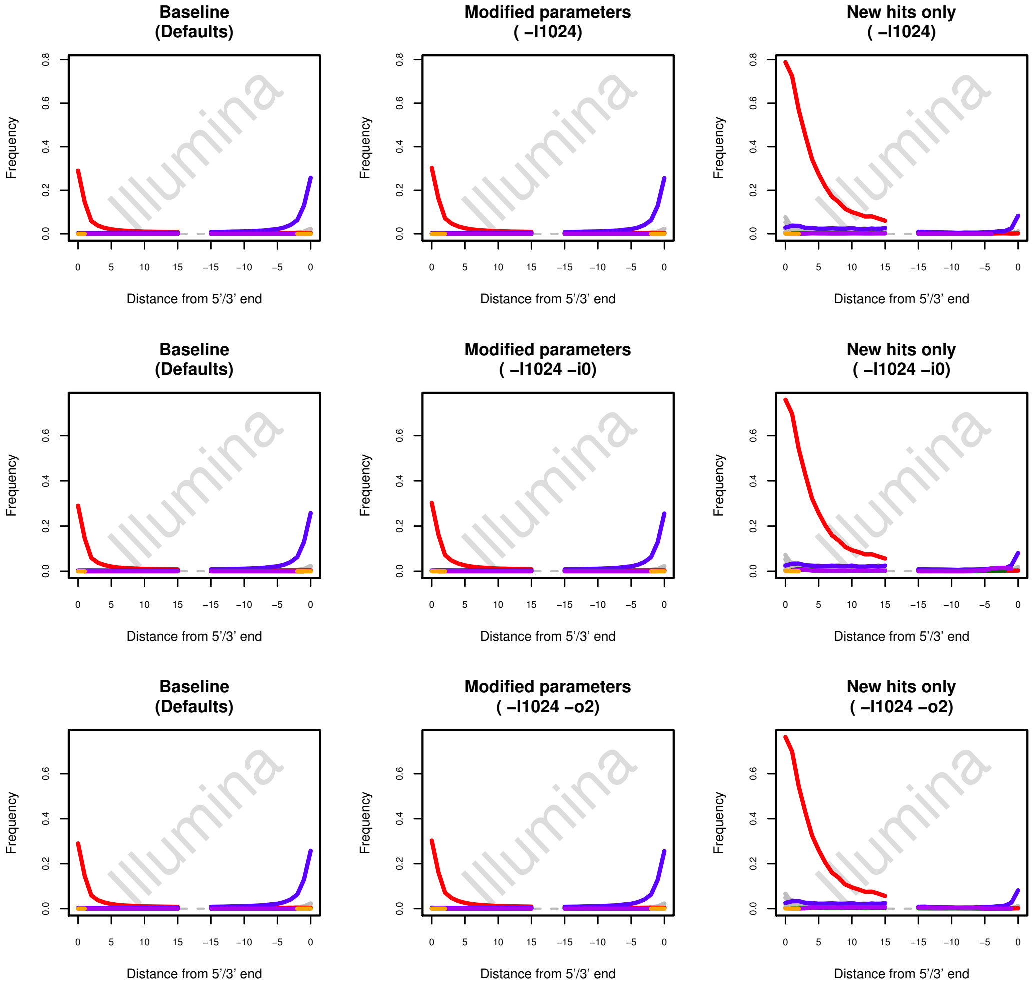

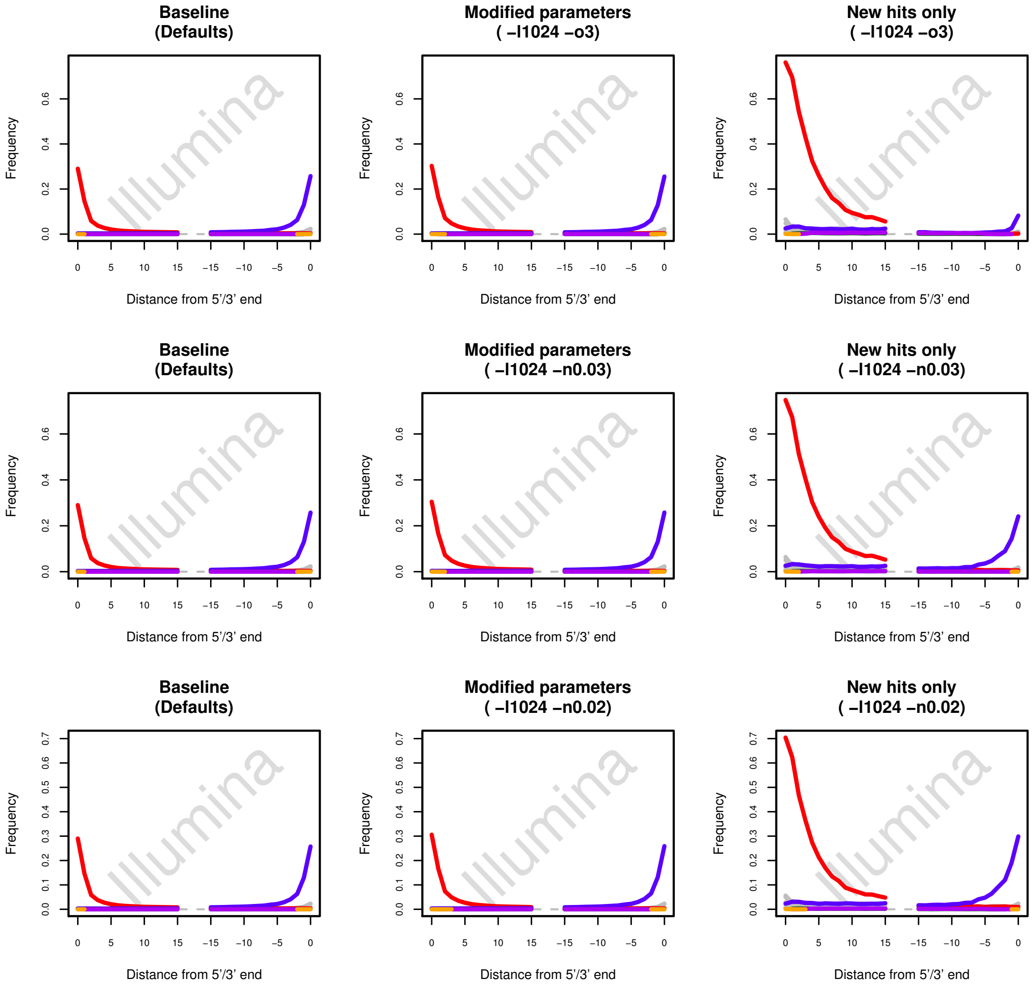

Nucleotide misincorporation patterns observed with Illumina reads and different combinations of BWA mapping parameters. Sequencing Illumina sequencing reads recovered from the sample showing infinite radiocarbon date were aligned using different combinations of mapping parameters using the BWA aligner. Reads were considered of high-quality when mapping uniquely to the EquCab2 genome but not against the human genome (assembly hg19) and showing mapping qualities of at least 25.mapping uniquely to the EquCab2 genome and showing mapping qualities of at least 25. In addition, positive hits were filtered for PCR duplicates (see Methods). Nucleotide misincorporation patterns observed when using the reads recovered from a first set of BWA parameters (left) are compared to those resulting from a second set of BWA parameters (middle). Nucleotide misincorporation patterns observed on the fraction of high-quality hits specifically identified with the second set of parameters are shown on the right. The specific set of mapping parameters is reported within parentheses with reference to options used in the bwa aln command line. Red: C→T. Blue: G→A. Pink: Insertions. Green: Deletions. Orange: Clipped bases. Grey: Other misincorporations.

Supplementary Figure 2

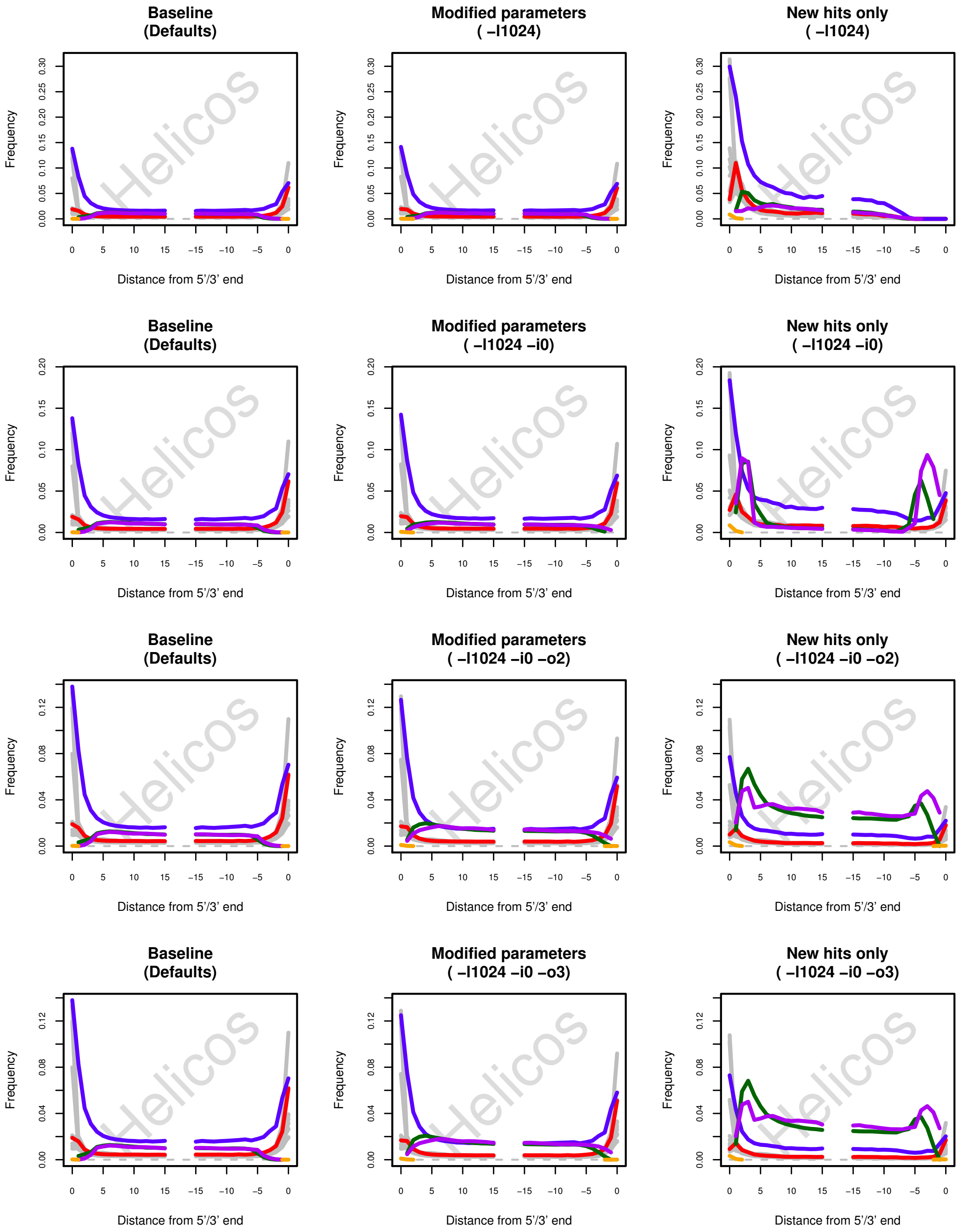

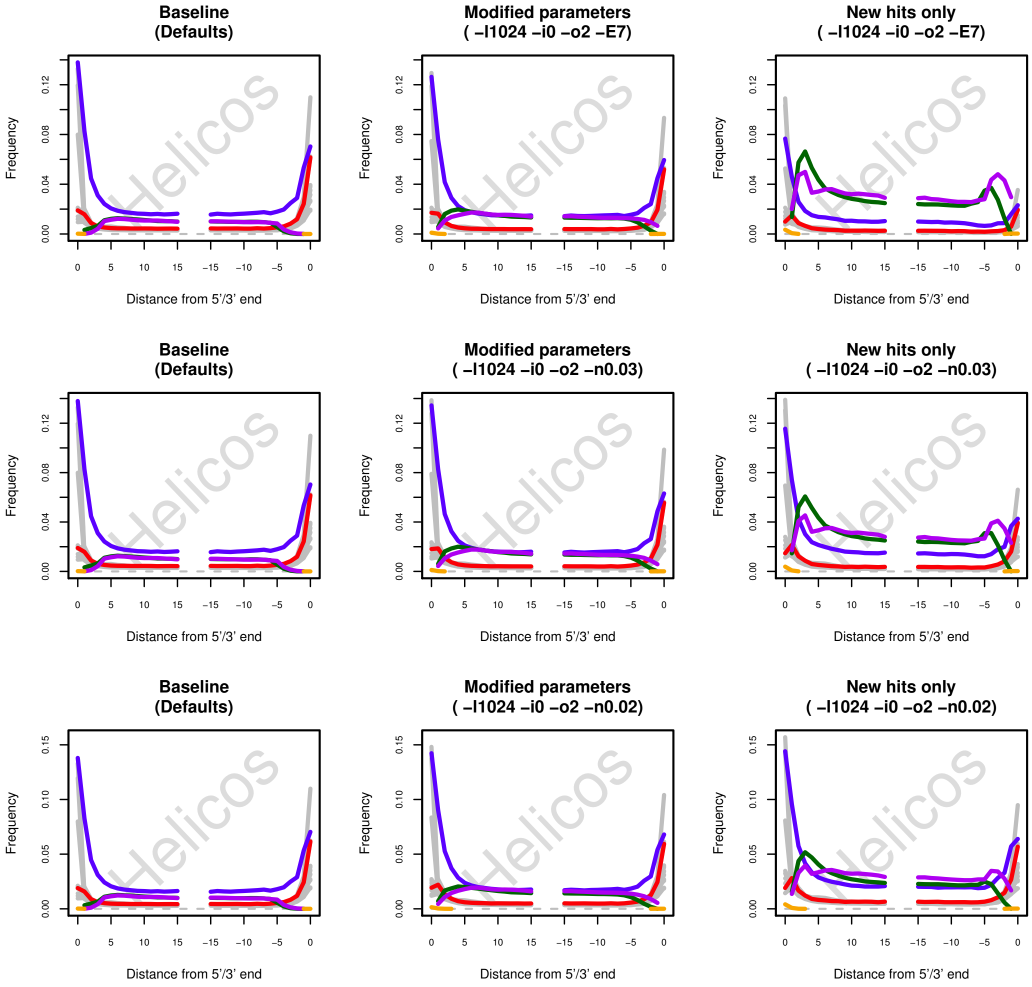

Nucleotide misincorporation patterns observed with Helicos reads and different combinations of BWA mapping parameters. Helicos Sequencing sequencing reads recovered from the sample showing infinite radiocarbon date were aligned using different combinations of mapping parameters using the BWA aligner. Reads were considered of high-quality when mapping uniquely to the EquCab2 genome but not against the human genome (assembly hg19) and showing mapping qualities of at least 25. mapping uniquely to the EquCab2 genome and showing mapping qualities of at least 25. Nucleotide misincorporation patterns observed when using the reads recovered from a first set of BWA parameters (left) are compared to those resulting from a second set of BWA parameters (middle). Nucleotide misincorporation patterns observed on the fraction of high-quality hits specifically identified with the second set of parameters are shown on the right. The specific set of mapping parameters is reported within parentheses with reference to options used in the bwa aln command line. Red: C→T. Blue: G→A. Pink: Insertions. Green: Deletions. Orange: Clipped bases. Grey: Other misincorporations.

Supplementary Figure 3

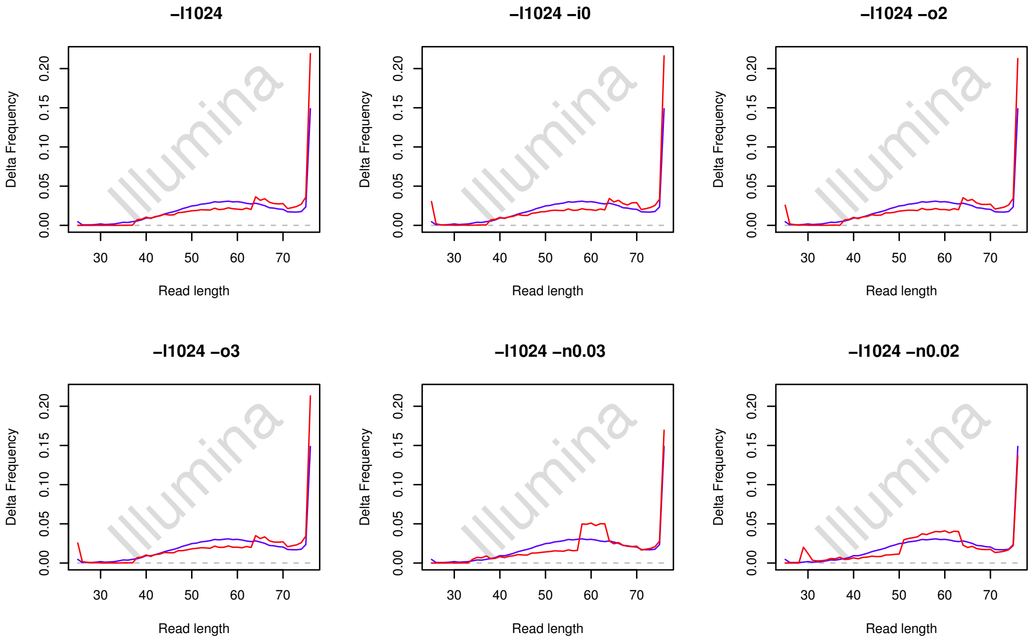

Length distribution of all horse hits recovered using default (blue) or modified (red) BWA parameters. Illumina sequencing reads recovered from the sample showing infinite radiocarbon date were aligned using different combinations of mapping parameters using the BWA aligner. Reads were considered of high-quality when mapping uniquely to the EquCab2 genome but not against the human genome (assembly hg19) and showing mapping qualities of at least 25. The size distribution of all high-quality reads identified with default parameters is plotted in blue while the size distribution of the new population of hits only identified with the new set of parameters is reported in red.

Supplementary Figure 4

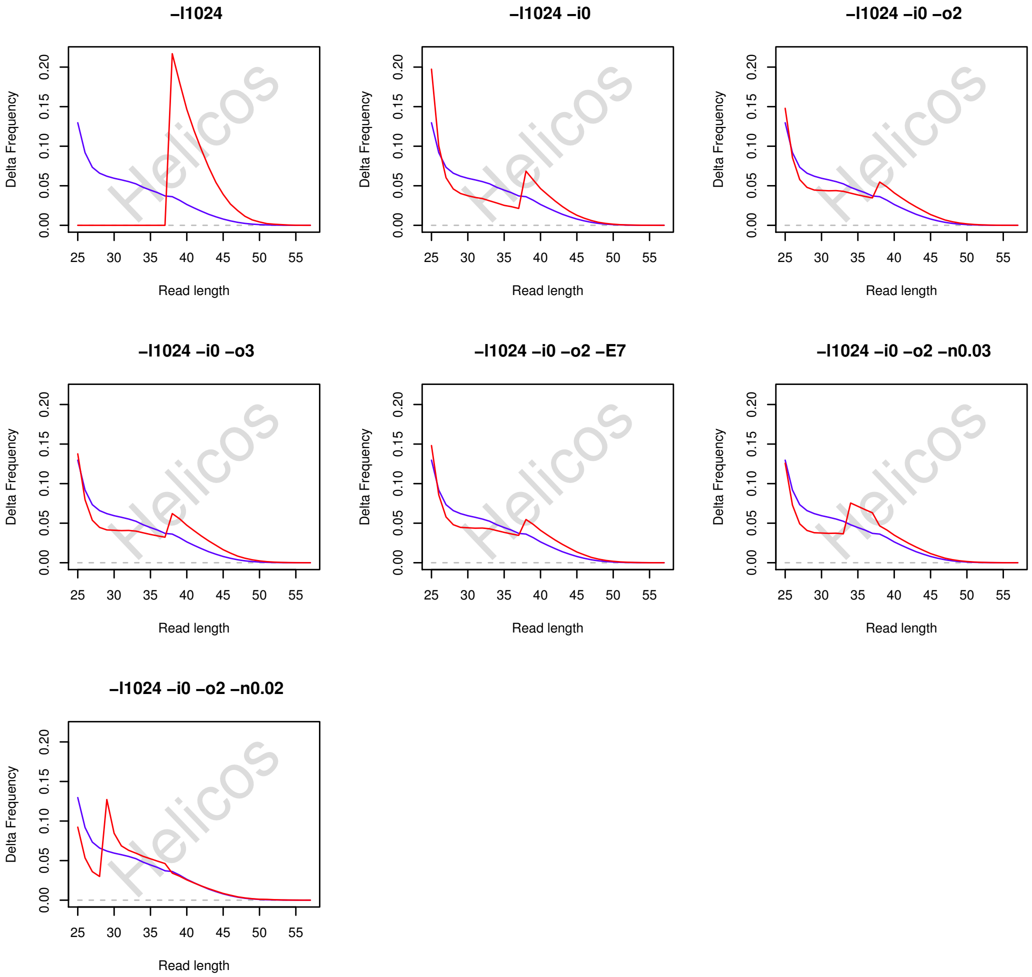

Length distribution of all horse hits recovered using default (blue) or modified (red) BWA parameters. Helicos sequencing reads recovered from the sample showing infinite radiocarbon date were aligned using different combinations of mapping parameters using the BWA aligner. Reads were considered of high-quality when mapping uniquely to the EquCab2 genome but not against the human genome (assembly hg19) and showing mapping qualities of at least 25. The size distribution of all high-quality reads identified with default parameters is plotted in blue while the size distribution of the new population of hits only identified with the new set of parameters is reported in red.

Supplementary Figure 5

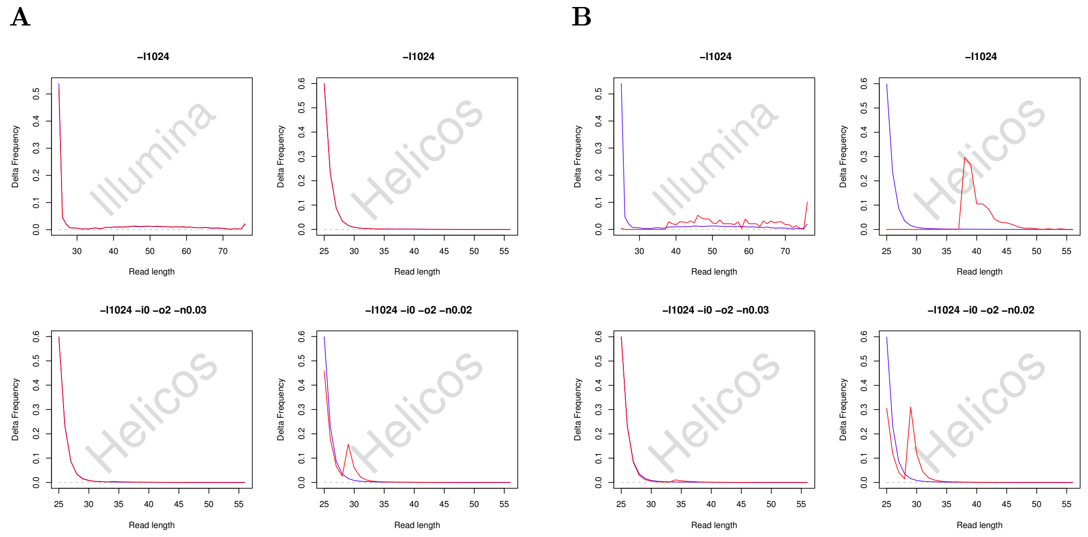

Length distribution of all hits recovered when mapping against the chicken genome and using default (blue) or modified (red) BWA parameters. Sequencing reads recovered from the sample showing infinite radiocarbon date were aligned using different combinations of mapping parameters using the BWA aligner. Reads were considered of high-quality when mapping uniquely to the galGal3 genome but not against the human genome (assembly hg19) and showing mapping qualities of at least 25. **Panel A:** The size distribution of all high-quality reads identified with default parameters is plotted in blue; the size distribution of all high-quality reads identified with modified parameters is plotted in red. **Panel B:** Same as in panel A, except for the size distribution reported in red which refers only to the new population of hits identified with the new set of parameters.

Supplementary Figure 6

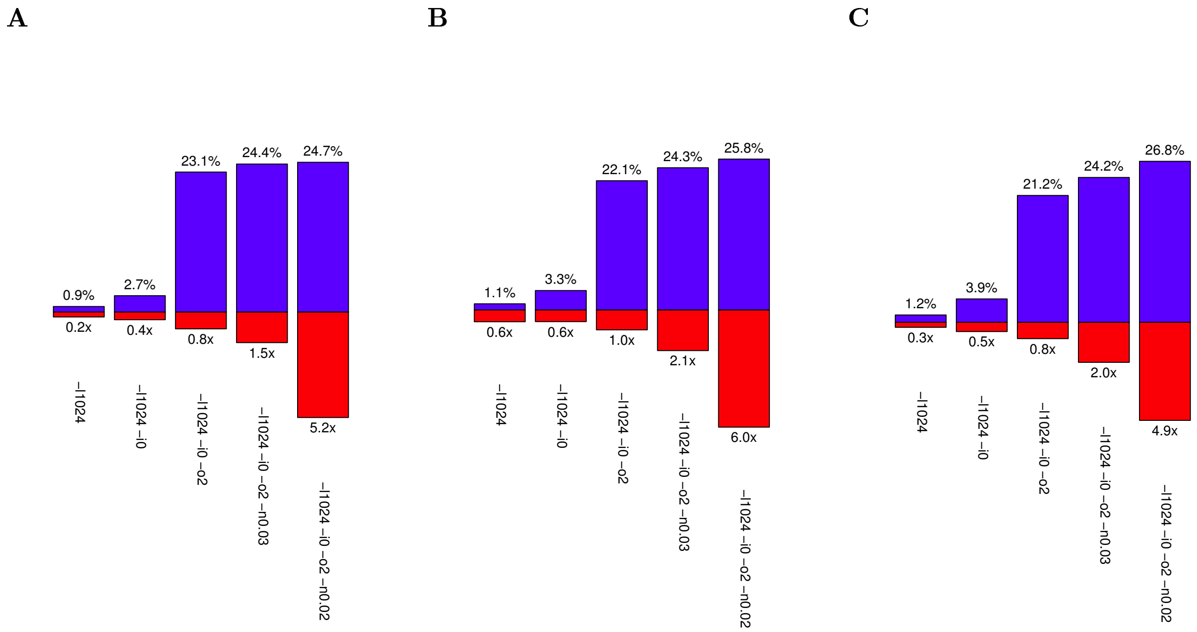

Exploring the effects of different sets of mapping parameters on BWA performance and runtime using simulated Helicos reads. One million of Helicos reads were simulated starting from random regions of the horse genome selected to fit the base composition bias specific to Helicos tSMS reads (see Methods). In addition, different sequencing errors were incorporated in the genomic regions selected using 1.5% of insertion per base, 3% of deletions per base and 0.5% (panel A), 1.0% (panel B) and 1.5% (panel C) for rates of substitutions per base. Reads were considered of high-quality when mapping uniquely to the EquCab2 genome but not against the human genome (assembly hg19) and showing mapping qualities of at least 25. Performance and runtime are estimated with reference to the standard default parameters.

Supplementary Figure 7

A

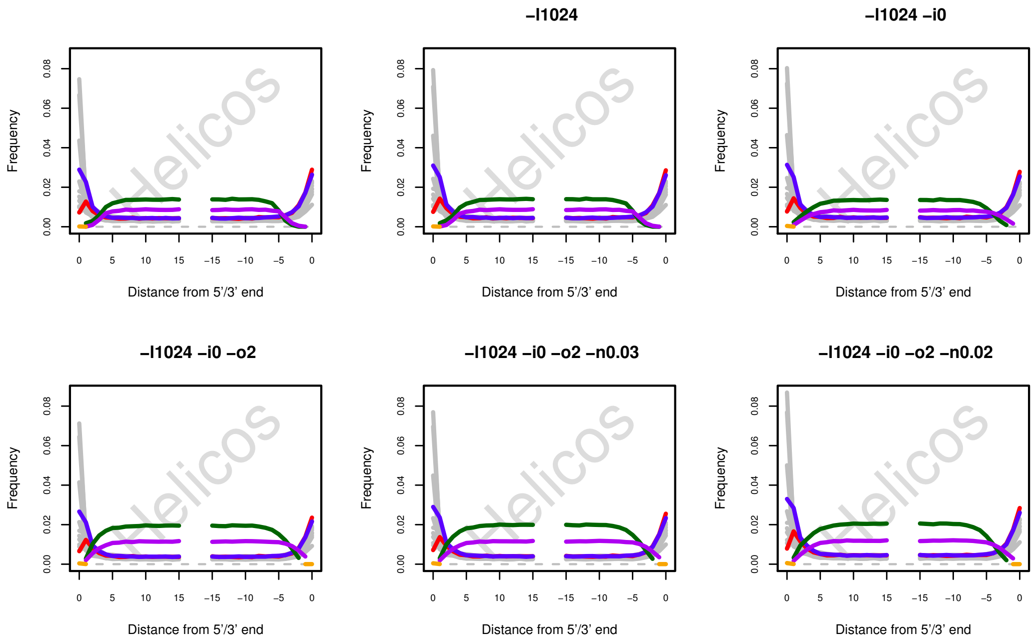

B

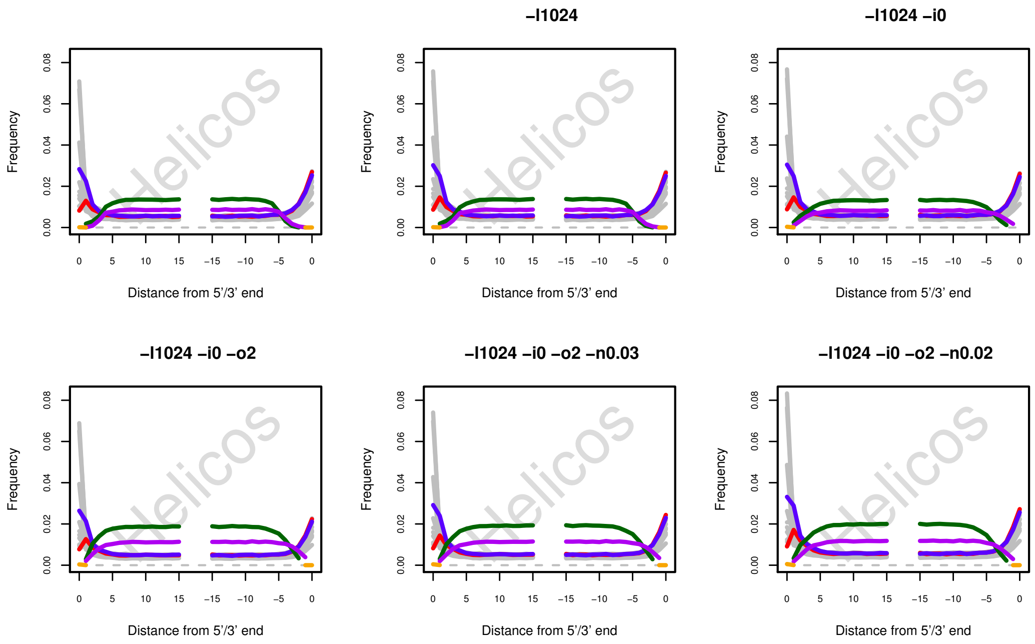

C

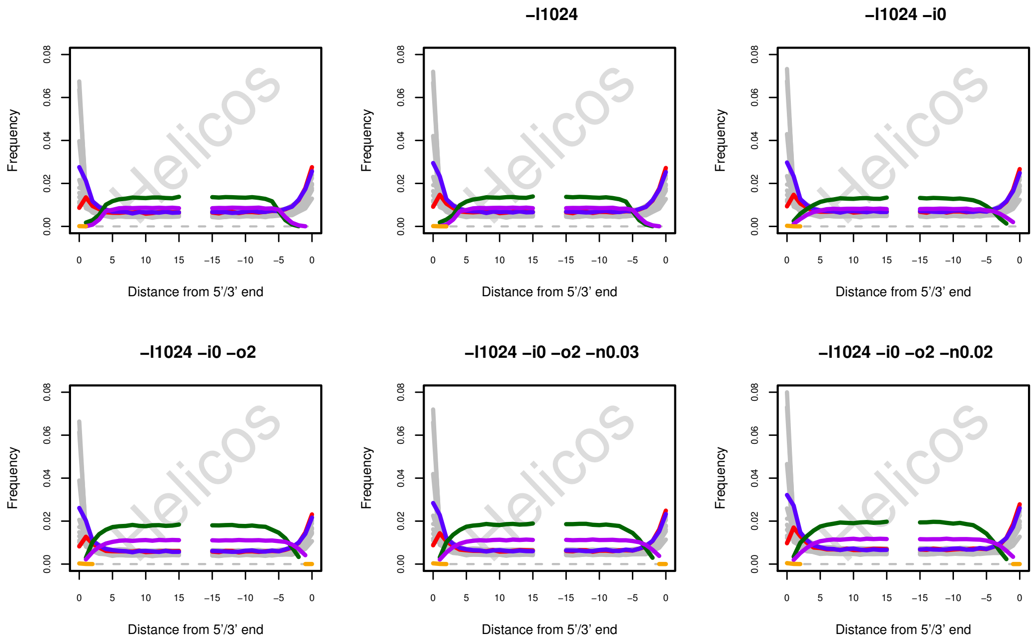

Nucleotide misincorporation patterns observed with simulated Helicos reads. One million of Helicos reads were simulated starting from random regions of the horse genome selected to fit the base composition bias specific to Helicos tSMS reads (see Methods). In addition, different sequencing errors were incorporated in the genomic regions selected using 1.5% of insertion per base, 3% of deletions per base and 0.5% (panel A), 1.0% (panel B) and 1.5% (panel C) for rates of substitutions per base. Reads were considered of high-quality when mapping uniquely to the EquCab2 genome but not against the human genome (assembly hg19) and showing mapping qualities of at least 25. Nucleotide misincorporation patterns are reported for the high-quality reads recovered using different sets of BWA parameters. The specific set of mapping parameters is reported in top of the graphs with reference to options used in the bwa aln command line.

Supplementary Figure 8

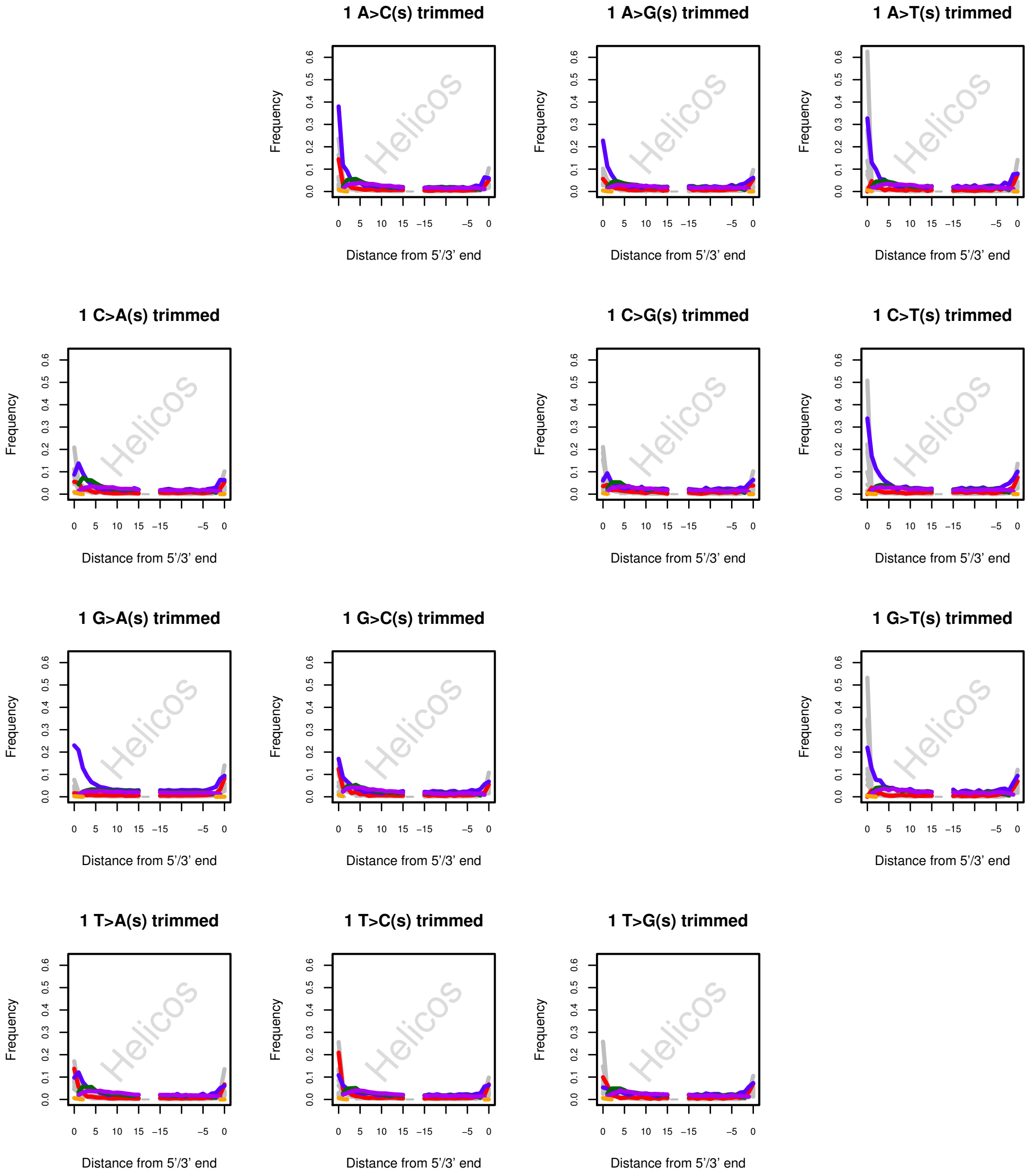

Nucleotide misincorporation patterns observed with Helicos reads following trimming of the first nucleotide sequenced. Helicos sequencing reads recovered from the sample showing infinite radiocarbon date were aligned using different combinations of mapping parameters using the BWA aligner. Reads were considered of high-quality when mapping uniquely to the equCab2 genome but not against the human genome (assembly hg19) and showing mapping qualities of at least 25. All reads that were not identified as high-quality following a first mapping procedure were trimmed for the first base at 5'-ends, remapped against equCab2 and hg19, and high-quality reads were identified as defined above. The base trimmed was then compared to the corresponding position in the horse reference genome in order to characterize which mismatch precluded mapping the first pass. Nucleotide misincorporation patterns are reported for the high-quality reads recovered for all classes of possible mismatches at the position of the trimmed base, which are reported above each individual graph.

Supplementary Figure 9

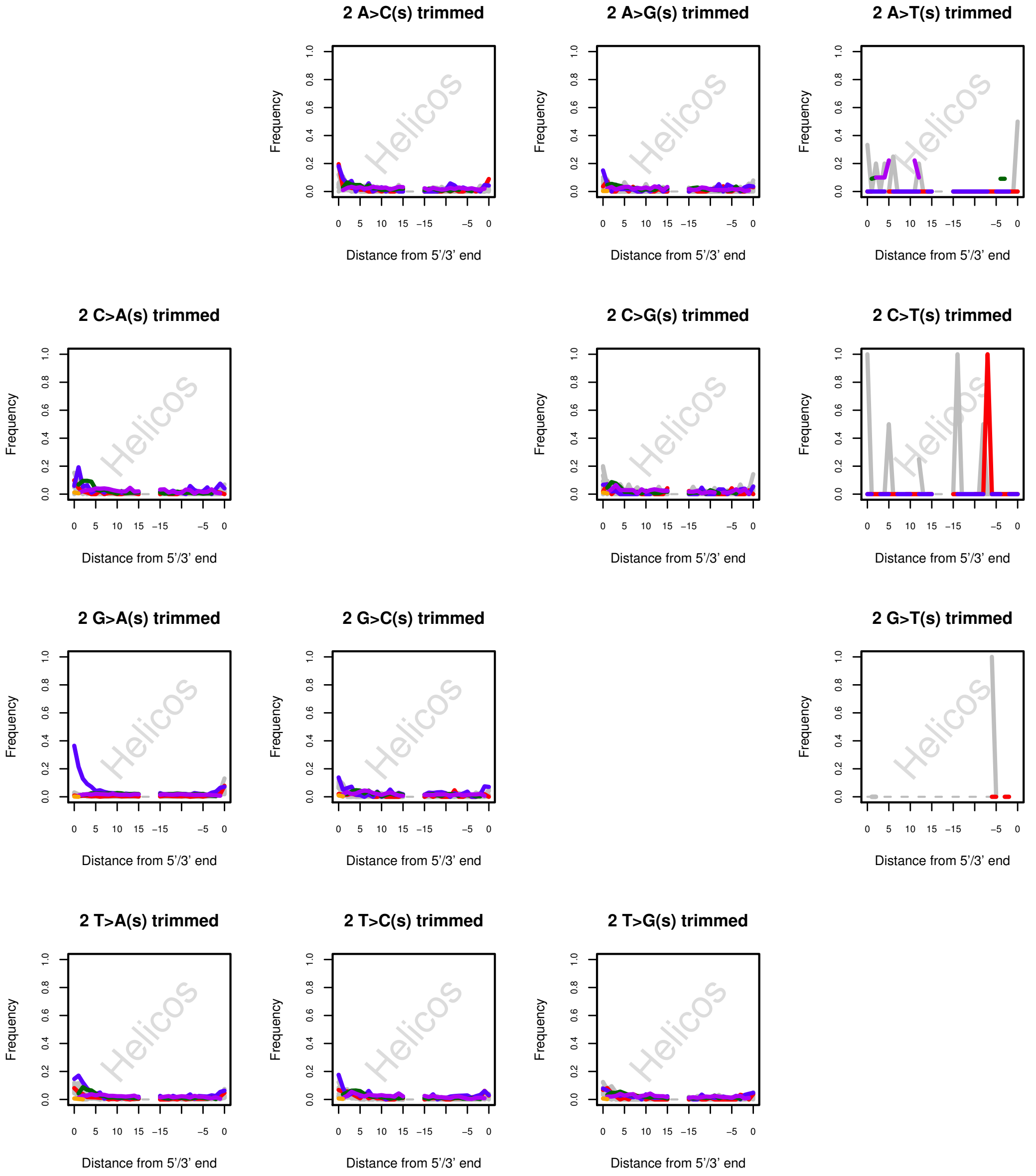

Nucleotide misincorporation patterns observed with Helicos reads following trimming of the first two nucleotides sequenced. Helicos sequencing reads recovered from the sample showing infinite radiocarbon date were aligned using different combinations of mapping parameters using the BWA aligner. Reads were considered of high-quality when mapping uniquely to the equCab2 genome but not against the human genome (assembly hg19) and *showing* mapping qualities of at least 25. All reads that were not identified as high-quality following a first mapping procedure were trimmed for the first two bases at 5'-ends, remapped against equCab2 and hg19, and high-quality reads were identified as defined above. The bases trimmed were then compared to the corresponding position in the horse reference genome in order to characterize which mismatch precluded mapping the first pass. We focused on situations where both bases showed the same type of mismatch. Nucleotide misincorporation patterns are reported for the high-quality reads recovered for all classes of possible mismatches at the position of the trimmed bases, which are reported above each individual graph.

Supplementary Figure 10

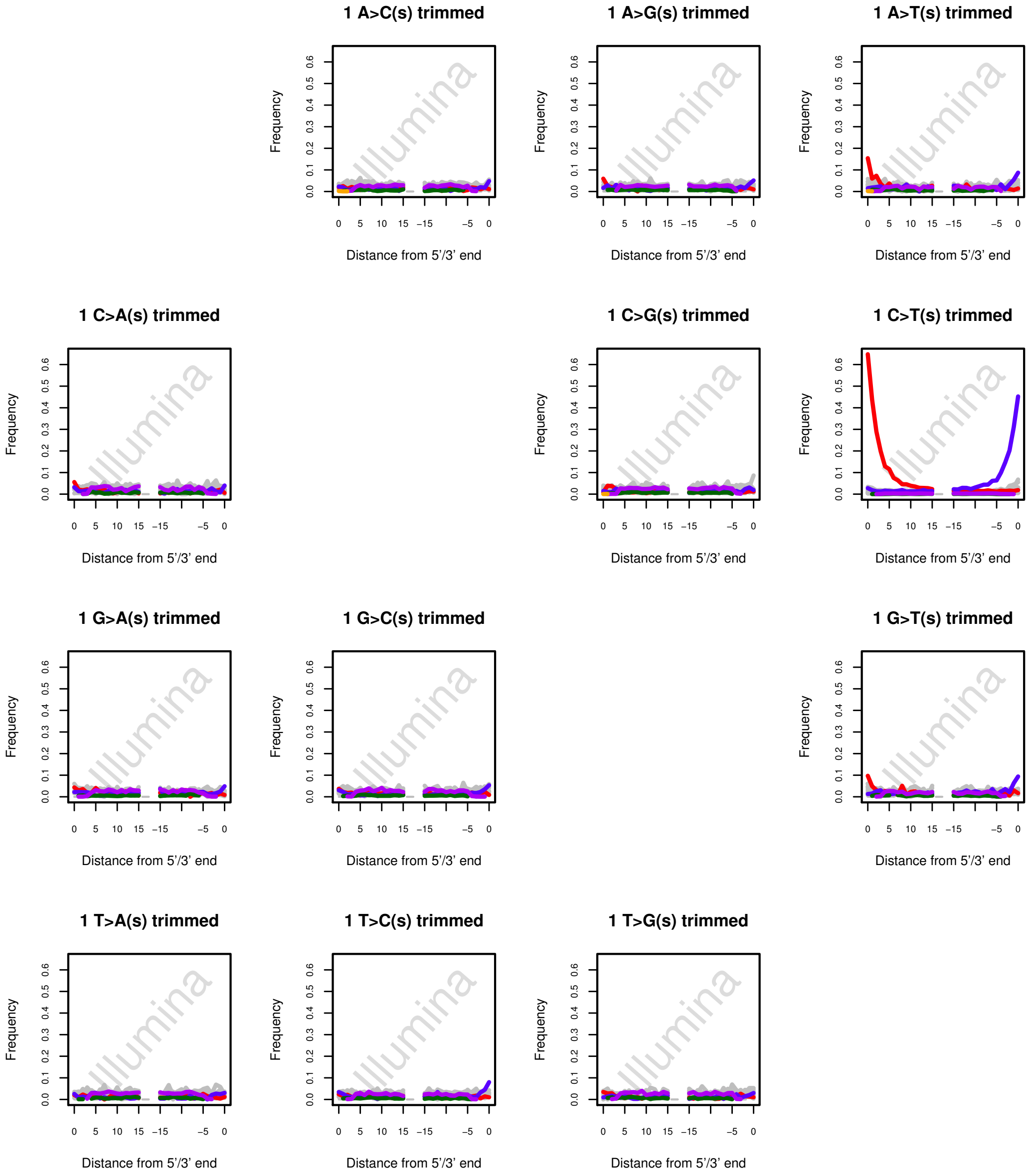

Nucleotide misincorporation patterns observed with Illumina reads following trimming of the first nucleotide sequenced. Illumina sequencing reads recovered from the sample showing infinite radiocarbon date were aligned using different combinations of mapping parameters using the BWA aligner. Reads were considered of high-quality when mapping uniquely to the equCab2 genome but not against the human genome (assembly hg19) and showing mapping qualities of at least 25. All reads that were not identified as high-quality following a first mapping procedure were trimmed for the first base at 5'-ends, remapped against equCab2 and hg19, and high-quality reads were identified as defined above. The base trimmed was then compared to the corresponding position in the horse reference genome in order to characterize which mismatch precluded mapping the first pass. Nucleotide misincorporation patterns are reported for the high-quality reads recovered for all classes of possible mismatches at the position of the trimmed base, which are reported above each individual graph.

Supplementary Figure 11

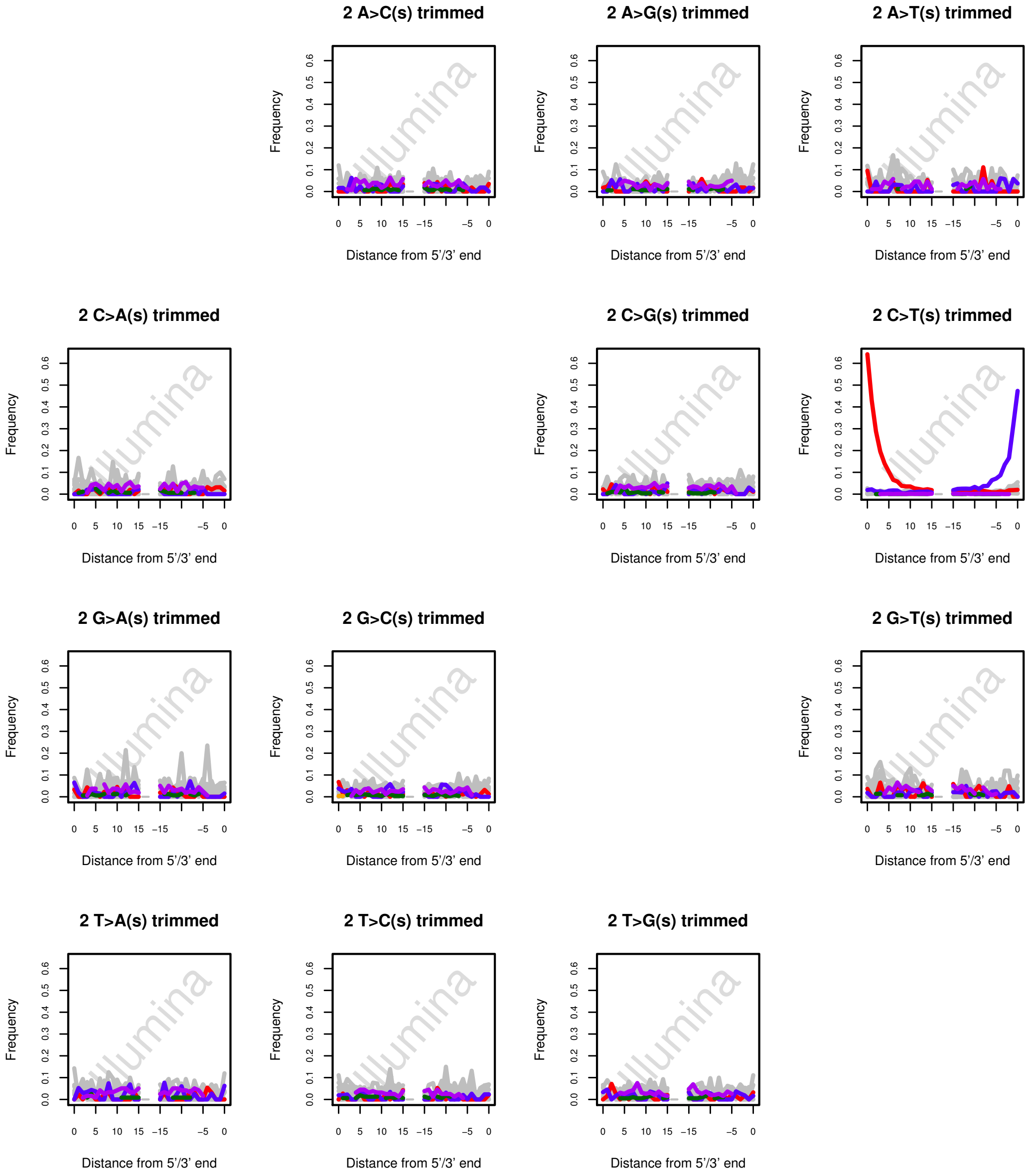

Nucleotide misincorporation patterns observed with Illumina reads following trimming of the first or the first two nucleotides sequenced. Illumina sequencing reads recovered from the sample showing infinite radiocarbon date were aligned using different combinations of mapping parameters using the BWA aligner. Reads were considered of high-quality when mapping uniquely to the equCab2 genome but not against the human genome (assembly hg19) and showing mapping qualities of at least 25. All reads that were not identified as high-quality following a first mapping procedure were trimmed for the first two bases at 5'-ends, remapped against equCab2 and hg19, and high-quality reads were identified as defined above. The bases trimmed were then compared to the corresponding position in the horse reference genome in order to characterize which mismatch precluded mapping the first pass. We focused on situations where both bases showed the same type of mismatch. Nucleotide misincorporation patterns are reported for the high-quality reads recovered for all classes of possible mismatches at the position of the trimmed bases, which are reported above each individual graph.

Supplementary Tables

Supplementary table 1

	0.0%		0.5%		1.0%		1.5%	
-l32	60.5	97.2	59.1	97.1	57.4	97.0	55.4	96.9
-l1024	61.1	97.2	59.7	97.1	58.1	97.1	56.2	96.9
-l1024 -i0	61.7	97.2	60.6	97.1	59.2	97.0	57.5	96.9
-l1024 -i0 -o2	75.7	97.4	73.3	97.3	70.6	97.2	67.5	97.0
-l1024 -i0 -o2 -n0.03	76.3	97.4	74.4	97.3	72.1	97.2	69.5	97.1
-l1024 -i0 -o2 -n0.02	76.6	97.3	75.5	97.2	73.9	97.1	71.9	96.9

BWA performance in mapping simulated Helicos reads to the bona-fide region of the horse genome. One million of Helicos reads were simulated starting from random regions of the horse genome selected to fit the base composition bias specific to Helicos tSMS reads (see Methods). In addition, different sequencing errors were incorporated in the genomic regions selected using 1.5% of insertion per base, 3% of deletions per base and 0.5%, 1.0% and 1.5% for rates of substitutions per base. Reads were considered of high-quality when mapping uniquely to the EquCab2 genome but not against the human genome (assembly hg19) and showing mapping qualities of at least 25. The fraction of high-quality reads identified is reported in the left column as percentage while the fraction of those that align in a genomic region located within 2bp of the true genomic positions is reported in the column, given underlying substitution rates per base of 0.0%, 0.5%, 1.0% and 1.5%.

Supplementary table 2

	Helicos		Illumina	
	1 NT	2 NTs	1 NT	2 NTs
A>C	0.365	0.4	0.045	0.051
A>G	0.198	0.215	0.034	0.039
A>T	0.148	0.148	0.05	0.049
C>A	0.317	0.331	0.017	0.022
C>G	0.152	0.166	0.029	0.034
C>T	0.182	0.182	0.531	0.638
G>A	0.904	1.069	0.023	0.027
G>C	0.289	0.304	0.044	0.049
G>T	0.146	0.146	0.057	0.06
T>A	0.45	0.472	0.016	0.019
T>C	0.436	0.478	0.05	0.059
T>G	0.212	0.221	0.026	0.029

Gains in high-quality hits identified following trimming of the first base or the first two bases at the 5'-ends of sequencing reads. Helicos and Illumina sequencing reads recovered from the sample showing infinite radiocarbon date were aligned using different combinations of mapping parameters using the BWA aligner. Reads were considered of high-quality when mapping uniquely to the equCab2 genome but not against the human genome (assembly hg19) and showing mapping qualities of at least 25. All reads that were not identified as high-quality following a first mapping procedure were trimmed for the first base or the first two bases at 5'-ends, remapped against equCab2 and hg19, and high-quality reads were identified as defined above. The bases trimmed were then compared to the corresponding position in the horse reference genome in order to characterize which types of mismatch precluded mapping the first pass. When two consecutive bases were trimmed, we focused on situations where both bases showed the same type of mismatch. The gains observed in the number of high-quality hits identified are reported relative to the number of hits identified using the optimized set of BWA parameters.

Supplementary table 3

		Horse	Human	Random
0.000%	Hits	762667	141706	11276
	Strict	6.6%	66.9%	20.6%
	Edit-distance	1.9%	66.2%	19.7%
	Δ	-71.1%	-1.1%	-4.6%
0.005%	Hits	743528	139638	11541
	Strict	6.4%	65.8%	19.4%
	Edit-distance	2.0%	65.0%	18.6%
	Δ	-69.0%	-1.2%	-4.2%
0.010%	Hits	721416	136409	11350
	Strict	6.3%	64.5%	20.7%
	Edit-distance	2.1%	63.7%	19.8%
	Δ	-66.8%	-1.2%	-4.4%
0.015%	Hits	694845	134311	11290
	Strict	6.2%	63.0%	20.2%
	Edit-distance	2.2%	62.2%	19.3%
	Δ	-64.7%	-1.3%	-4.3%

Gains in high-quality hits identified following trimming of the first base or the first two bases at the 5'-ends of sequencing reads. One million of Helicos reads were simulated starting from random regions of the horse genome selected to fit the base composition bias specific to Helicos tSMS reads (see Methods). In addition, reads from the human genome, or totally random reads were simulated. Different sequencing errors were incorporated in the genomic regions selected using 1.5% of insertion per base, 3% of deletions per base and 0.0%, 0.5%, 1.0% and 1.5% for rates of substitutions per base. In a first mapping procedure (procedure 1, Strict), reads were considered of high-quality when mapping uniquely to the EquCab2 genome but not against the human genome (assembly hg19). In a second mapping procedure (procedure 2, Edit distance), reads were considered of high-quality when mapping uniquely to the EquCab2 genome as long as no hit was observed against the human genome (assembly hg19) or as long as the edit distance to the horse genome was lower than the edit distance to the human genome. High-quality reads presented minimal mapping qualities of 25. Both mapping procedures were performed on the different sets of simulated reads and for a series of different mapping parameters. Both mapping procedures were performed on the different sets of simulated reads, aligned using the final set of suggested parameters for Helicos data. The total number of hits from each source, as well as the fraction of these hits filtered with each method (Strict, or Edit-distance), and the relative change in the number hits filtered (Δ) is reported.
